# Polar Ferromagnet Induced by Fluorine Positioning in Isomeric Layered Copper Halide Perovskites

**DOI:** 10.1021/acs.inorgchem.1c03726

**Published:** 2022-02-09

**Authors:** Ceng Han, Jason A. McNulty, Alasdair J. Bradford, Alexandra M. Z. Slawin, Finlay D. Morrison, Stephen L. Lee, Philip Lightfoot

**Affiliations:** † School of Chemistry and EaStChem, University of St Andrews, St Andrews KY16 9ST, U.K.; ‡ School of Physics, University of St Andrews, St Andrews, Fife KY16 9SS, U.K.

## Abstract

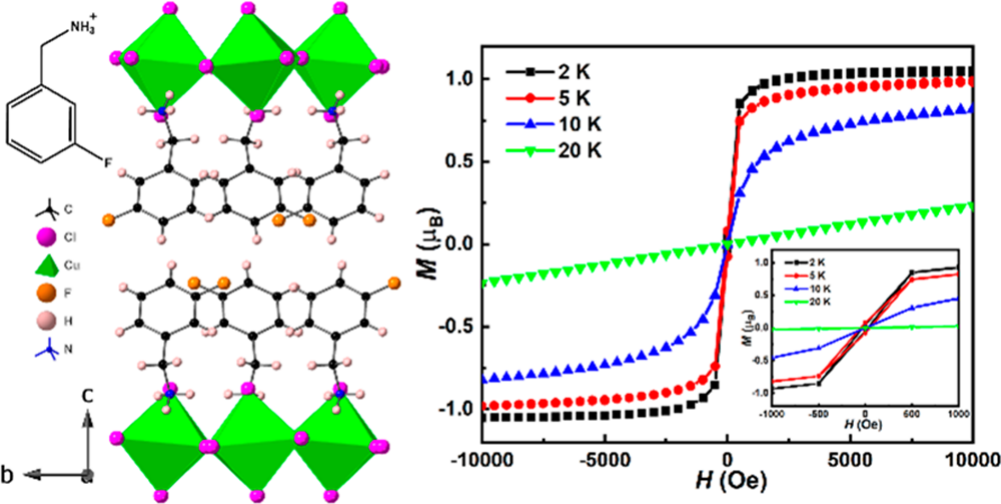

We present the influence of positional isomerism on the crystal structure of fluorobenzylammonium copper(II) chloride perovskites A_2_CuCl_4_ by incorporating *ortho-*, *meta*-, and *para*-fluorine substitution in the benzylamine structure. Two-dimensional (2D) polar ferromagnet (3-FbaH)_2_CuCl_4_ (3-FbaH^+^ = 3-fluorobenzylammonium) is successfully obtained, which crystallizes in a polar orthorhombic space group *Pca*2_1_ at room temperature. In contrast, both (2-FbaH)_2_CuCl_4_ (2-FbaH^+^ = 2-fluorobenzylammonium) and (4-FbaH)_2_CuCl_4_ (4-FbaH^+^ = 4-fluorobenzylammonium) crystallize in centrosymmetric space groups *P*2_1_/*c* and *Pnma* at room temperature, respectively, displaying significant differences in crystal structures. These differences indicate that the position of the fluorine atom is a driver for the polar behavior in (3-FbaH)_2_CuCl_4_. Preliminary magnetic measurements confirm that these three perovskites possess dominant ferromagnetic interactions within the inorganic [CuCl_4_]_∞_ layers. Therefore, (3-FbaH)_2_CuCl_4_ is a polar ferromagnet, with potential as a type I multiferroic. This work is expected to promote further development of high-performance 2D copper(II) halide perovskite multiferroic materials.

## Abstract 2

We present the influence of positional isomerism on the crystal structure of fluorobenzylammonium copper(II) chloride perovskites A_2_CuCl_4_ by incorporating *ortho*-, *meta*-, and *para*-fluorine substitution in the benzylamine structure. All three compounds display ferromagnetic interactions. *Meta-*F-substitution in (3-FbaH)_2_CuCl_4_ leads to a polar structure, and therefore it is a polar ferromagnet.

## Introduction

Two-dimensional (2D) organic–inorganic hybrid perovskites are evolving into an important class of materials displaying a diverse range of physical properties such as photovoltaic activity, luminescence, and ferroelectricity.^1−4^ Among the known families of two-dimensional layered perovskites, two conventional families are the Dion–Jacobson (DJ)^5^ and Ruddlesden–Popper (RP)^6^ phases, which are commonly defined in terms of their generic stoichiometries ABX_4_ and A_2_BX_4_, respectively, for examples with single octahedral layers. The structure of these compounds can be regarded as derived by slicing the cubic perovskite aristotype ABX_3_ along vertices of the BX_6_ octahedra and inserting additional moieties between these layers. An alternative definition of these generic families lies in the relative “staggering” of adjacent perovskite-like layers. In purely inorganic systems, the DJ family has “eclipsed” layers (i.e., (0,0) relative shift in the *ab*-plane of the idealized tetragonal unit cell), whereas the RP family exhibits a (1/2,1/2) shift. There is a third, intermediate case displaying a (1/2,0) shift, which has been designated DJ2.^7^ However, in the case of hybrid perovskites, the huge variation of interlayer ion sizes and shapes seriously affects the final structure, resulting in a much more complex array of possible “shifts” of adjacent layers, often resulting in adjacent layers being neither “fully eclipsed” (DJ-like) nor “fully staggered” (RP-like).^8−11^ Therefore, recent papers have suggested a more flexible structural classification of hybrid layered perovskites, beyond the simple “DJ or RP” types, according to the relative shifts of neighboring inorganic layers and regardless of the stoichiometries ABX_4_ and A_2_BX_4_.^12−14^ Introducing organic molecules with halogen substitution into the hybrid perovskite structure has been widely investigated to control the crystal symmetry, which has been proved to be a powerful approach to modify the physical and chemical properties.^15−18^

Recently copper-based halide perovskites with Jahn–Teller (J–T) distortion of the 3d^9^ ion Cu^2+^ have been widely explored as a platform for developing new multifunctional materials due to their interesting thermochromism, ferromagnetism, and ferroelectricity.^19−22^ To date, few copper-based halide perovskites with halogen-substituted organic molecule spacers have been prepared. 2D layered perovskites (CEA)_2_CuCl_4_ (CEA = 2-chloroethylammonium and (BEA)_2_CuCl_4_ (BEA = 2-bromoethylammonium) with chlorine and bromine substitution in ethylamine exhibit reversible and irreversible thermochromism, which can be modulated by incorporating organic cations.^23^ The introduction of two fluorine substituents in the centrosymmetric (CBA)_2_CuCl_4_ (CBA = cyclobutylammonium) structure results in a polar structure (DF-CBA)_2_CuCl_4_ (DF-CBA = 3,3-difluorocyclobutylammonium) with a ferroelectric phase transition temperature of 380 K.^24^

Despite considerable research, 2D copper layered perovskites with monofluorine-substituted organic spacers are still relatively unknown. Herein, for the first time, we present three new 2D hybrid layered copper(II) chloride perovskites, (2-FbaH)_2_CuCl_4_ (2-FbaH^+^ = 2-fluorobenzylammonium), (3-FbaH)_2_CuCl_4_ (3-FbaH^+^ = 3-fluorobenzylammonium), and (4-FbaH)_2_CuCl_4_ (4-FbaH^+^ = 4-fluorobenzylammonium), by incorporating *ortho*-, *meta*-, and *para*-fluorine substitution into the benzylamine structure. The monofluorine substitution at various positions in the benzylamine structure results in significant differences in crystal symmetry. Here we show that (3-FbaH)_2_CuCl_4_ crystallizes in a polar orthorhombic space group *Pca*2_1_ at room temperature. In contrast, both isomers (2-FbaH)_2_CuCl_4_ and (4-FbaH)_2_CuCl_4_ crystallize in centrosymmetric space groups at room temperature. Magnetic measurements confirm that all three perovskites exhibit ferromagnetism, indicating the potential multiferroic behavior of the polar ferromagnet (3-FbaH)_2_CuCl_4_.

## Experimental Section

### Materials

Copper(II) chloride anhydrous (CuCl_2_, 98%), hydrochloric acid (HCl, 36%, w/w, aqueous solution), and ethanol absolute (C_2_H_5_OH, 99.99%) were purchased from Alfa Aesar. 2-Fluorobenzylamine (C_7_H_8_NF, 97%), 3-fluorobenzylamine (C_7_H_8_NF, 97%), and 4-fluorobenzylamine (C_7_H_8_NF, 98%) were purchased from Fluorochem. All chemicals were directly used without further purification.

### Synthesis

The compounds (2-FbaH)_2_CuCl_4_, (3-FbaH)_2_CuCl_4_, and (4-FbaH)_2_CuCl_4_ were crystallized by a slow evaporation method.

For (2-FbaH)_2_CuCl_4_ (C_14_H_18_F_2_N_2_CuCl_4_), CuCl_2_ (268.9 mg, 2 mmol) was dissolved in concentrated HCl (5 mL) and ethanol (2 mL) with moderate heating. Once fully dissolved, 2-fluorobenzylamine (0.48 mL, 4 mmol) was added, and the solution was allowed to cool. By cooling overnight, green plate-shaped crystals were obtained. Elemental Anal. Calcd (%) for (2-FbaH)_2_CuCl_4_: C, 36.74; H, 3.96; N, 6.12. Found: C, 36.82; H, 3.97; N, 5.99.

For (3-FbaH)_2_CuCl_4_ (C_14_H_18_F_2_N_2_CuCl_4_), CuCl_2_ (134.45 mg, 1 mmol) was dissolved in concentrated HCl (20 mL) and ethanol (20 mL) with slow stirring and moderate heating. Once fully dissolved, 3-fluorobenzylamine (0.24 mL, 2 mmol) was added. The produced precipitates were dissolved by adding excess concentrated HCl to get a clear solution. By naturally cooling the solvent for a few hours, yellow plate-shaped crystals were obtained. Elemental Anal. Calcd (%) for (3-FbaH)_2_CuCl_4_: C, 36.74; H, 3.96; N, 6.12. Found: C, 36.88; H, 3.91; N, 5.97.

For (4-FbaH)_2_CuCl_4_ (C_14_H_18_F_2_N_2_CuCl_4_), CuCl_2_ (134.45 mg, 1 mmol) was dissolved in concentrated HCl (20 mL) and ethanol (20 mL) with moderate heating. Once fully dissolved, 4-fluorobenzylamine (0.24 mL, 2 mmol) was added. The produced precipitates were dissolved by adding excess concentrated HCl to get a clear solution. By cooling for a few hours, yellow plate-shaped crystals were obtained. Elemental Anal. Calcd (%) for (4-FbaH)_2_CuCl_4_: C, 36.74; H, 3.96; N, 6.12. Found: C, 36.88; H, 3.89; N, 5.95.

### Characterization

#### Single Crystal X-ray Diffraction

Single crystal X-ray diffraction data were collected on a Rigaku XtaLAB P200 diffractometer at 93 K and on a Rigaku SCX Mini diffractometer at 173 and 298 K using Mo Kα radiation (λ = 0.71075 Å). The data were processed by using Rigaku CrystalClear software.^25^ Crystal structures were solved by using structure solution program SHELXT,^26^ and full-matrix least-squares refinements on *F*^2^ were performed by using SHELXL-2018/3^26^ incorporated in the WinGX program.^27^ Absorption corrections were conducted empirically from equivalent reflections according to multiscans using CrystalClear.^25^ All the hydrogen atoms were treated as riding atoms, and all non-H atoms were refined anisotropically.

#### Powder X-ray Diffraction (PXRD)

Powder X-ray diffraction data were measured on a PANalytical EMPYREAN diffractometer using Cu Kα_1_ (λ = 1.5406 Å) radiation at ambient temperature. The data were collected in the range of 3°–70° for 1 h to confirm the purity of each sample. Variable-temperature PXRD was run on a PANalytical EMPYREAN diffractometer using Mo Kα radiation, from room temperature to 433 K.

#### Thermogravimetric Analyses (TGA)

TGA data were collected on a STA-780 instrument between 293 and 523 K at a heating rate of 5 K min^–1^ under flowing N_2_.

#### Electrical Characterization

Dielectric and impedance spectroscopy measurements were made on pellets ca. 1 mm thick and 10 mm in diameter formed by uniaxially pressing powder under a load of 2 tons. Silver conductive paste was applied to the opposing pellet faces and allowed to dry at 373 K. The data were recorded over the frequency range 100 Hz and 10 MHz at 1 K increments at a heating/cooling rate of 1 K min^–1^ between 298 and 473 K by using a closed cycle cryocooler and furnace.

#### Magnetic Measurements

The magnetic measurements were performed on a Quantum Design (MPMS XL) SQUID magnetometer. Data were collected by cooling a known mass of material within a 100 Oe field at 10 K intervals between 300 and 2 K.

## Results and Discussion

### Crystal Structures

The single crystal X-ray structures suggest no phase transitions in the regime 93 < *T* < 298 K, so the crystallographic details will be discussed based on the structures at 298 K. Details of the structures at 93 and 173 K are provided in the Supporting Information. Crystallographic parameters for all three compounds at 298 K are given in Table 1 and selected geometrical parameters in Table 2.

**Table 1:** Crystal and Refinement Data for (2-FbaH)_2_CuCl_4_, (3-FbaH)_2_CuCl_4_, and (4-FbaH)_2_CuCl_4_ at 298 K

compound	(2-FbaH)_2_CuCl_4_	(3-FbaH)_2_CuCl_4_	(4-FbaH)_2_CuCl_4_
formula	C_14_H_18_F_2_N_2_CuCl_4_	C_14_H_18_F_2_N_2_CuCl_4_	C_14_H_18_F_2_N_2_CuCl_4_
formula weight	457.64	457.64	457.64
color/habit	green/platelet	yellow/platelet	yellow/platelet
crystal size (mm^3^)	0.25 × 0.25 × 0.08	0.33 × 0.32 × 0.15	0.17 × 0.10 × 0.06
crystal system	monoclinic	orthorhombic	orthorhombic
space group	*P*2_1_/*c*	*Pca*2_1_	*Pnma*
*a* (Å)	5.3813(4)	7.5770(5)	10.6126(7)
*b* (Å)	5.1660(3)	7.2883(5)	31.3460(2)
*c* (Å)	31.817(2)	32.525(2)	5.2326(4)
α (deg)	90	90	90
β (deg)	90.975(4)	90	90
γ (deg)	90	90	90
*V* (Å^3^)	884.38(10)	1796.1(2)	1740.69(18)
*Z*	2	4	4
ρ_calc_ (g/cm^3^)	1.719	1.692	1.746
μ (mm^–1^)	1.856	1.828	1.886
*F*(000)	462	924	924
no. of reflns collected	7019	16073	16385
independent reflns	1971	4014	2030
	[*R*(int) = 0.0352]	[*R*(int) = 0.0421]	[*R*(int) = 0.0673]
goodness of fit	1.052	1.063	0.998
final *R* indices (*I* > 2σ(*I*))	*R*_1_ = 0.0335	*R*_1_ = 0.0258	*R*_1_ = 0.0304
	*wR*_2_ = 0.0777	*wR*_2_ = 0.0646	*wR*_2_ = 0.0711
largest diff peak/hole (e Å^–3^)	0.409/–0.344	0.254/–0.342	0.293/–0.285

**Table 2:** Cu–Cl Bond Lengths and Cu–Cl–Cu Bond Angles for the Three Structures at 298 K

	(2-FbaH)_2_CuCl_4_	(3-FbaH)_2_CuCl_4_	(4-FbaH)_2_CuCl_4_
*R*_S_ (Å)	2.301(2)	2.292(7)	2.298(2)
		2.297(7)	
*R*_L_ (Å)	3.012(2)	3.001(8)	2.978(7)
		2.959(8)	
*R*_Z_ (Å)	2.277(2)	2.294(11)	2.280(6)
		2.290(11)	
(2/√3)[*R*_L_ – (*R*_S_ + *R*_Z_)/2] (Å)^35^	0.835	0.793	0.796
Cu–Cl–Cu (deg)	162.3(4)–169.0(4)	169.5(3)–171.3(3)	174.3(4)–175.3(6)

The powder X-ray diffraction (PXRD) patterns of (2-FbaH)_2_CuCl_4_, (3-FbaH)_2_CuCl_4_, and (4-FbaH)_2_CuCl_4_ all exhibit similar features to those calculated based on their single crystal structures (Figure S1). Rietveld refinements were performed to confirm the purity of the as-synthesized crystals (Figure S2). Thermogravimetric analysis (TGA) measurements indicate that these compounds show superior thermal stability, with decomposition temperatures up to 450, 462, and 464 K for (2-FbaH)_2_CuCl_4_, (3-FbaH)_2_CuCl_4_, and (4-FbaH)_2_CuCl_4_, respectively (Figure S3).

**Figure 1:**
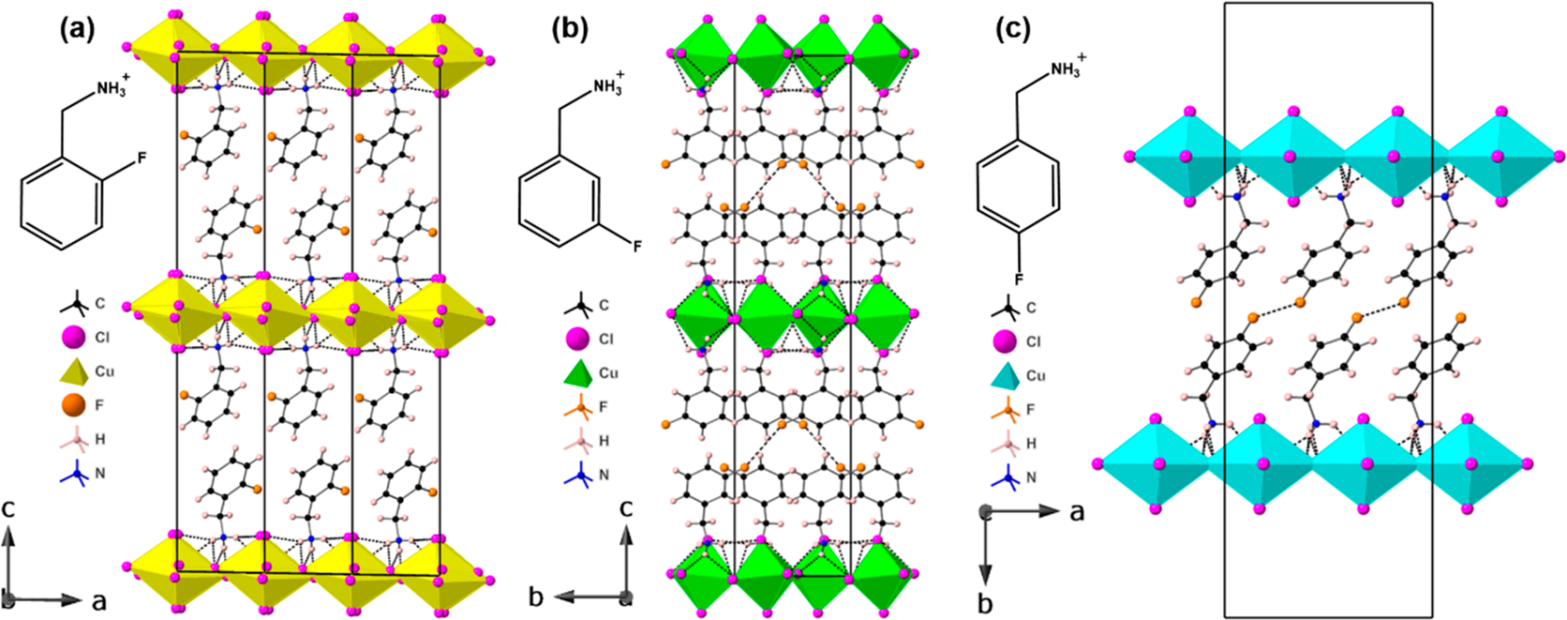
Crystal structures of (a) (2-FbaH)_2_CuCl_4_, (b) (3-FbaH)_2_CuCl_4_, and (c) (4-FbaH)_2_CuCl_4_ at 298 K parallel to the layer direction.

The crystal structures of (2-FbaH)_2_CuCl_4_, (3-FbaH)_2_CuCl_4_, and (4-FbaH)_2_CuCl_4_ can be described as 2D layered perovskite structures, with single layers of corner-shared CuCl_6_ octahedra forming a layer stoichiometry [CuCl_4_]_∞_, which are separated by a double layer of the protonated fluorobenzylammonium moieties (Figure 1), as found in their non-fluorinated counterpart (BaH)_2_CuCl_4_ (BaH^+^ = benzylammonium).^28^ We will first discuss general features and differences of the three crystal structures from the perspective of the inorganic “framework”, as these can be discussed systematically based on the known principles of perovskite crystallography. It is often helpful to rationalize the unit cell metrics of layered perovskites in relation to the idealized parent DJ or RP phases, which have tetragonal symmetry as well as space groups *P*4/*mmm* and *I*4/*mmm* and incorporate one and two [CuCl_4_]_∞_ layers per unit cell, respectively. The parent benzylammonium compound (BaH)_2_CuCl_4_ adopts the triclinic space group *P*-1 and has unit cell metrics at 293 K, *a* = 10.501(2) Å, *b* = 10.576(2) Å, *c* = 16.115(3) Å, α = 98.00(3), β = 99.33(3)°, and γ = 90.06(3)°. Thus, this unit cell can be regarded as a 2*a*_DJ_ × 2*a*_DJ_ × *c*_DJ_ supercell of the parent ABX_4_ DJ structure. In contrast, the monofluorine substitution on various positions of the benzylamine molecule results in significant differences in crystal symmetry, and each of the present fluorinated derivatives crystallizes with two [CuCl_4_]_∞_ layers per unit cell, rather than one. In that sense, they may be better regarded as derived from the RP rather than the DJ parent. The data in Table 1 reveal that the corresponding supercell metrics for the present compounds are *a*_RP_ × *a*_RP_ × *c*_RP_ for (2-FbaH)_2_CuCl_4_, √2*a*_RP_ × √2*a*_RP_ × *c*_RP_ for (3-FbaH)_2_CuCl_4_, and 2*a*_RP_ × *c*_RP_ × *a*_RP_ in the case of (4-FbaH)_2_CuCl_4_. Further details of the underlying distortions are addressed below.

**Figure 2:**
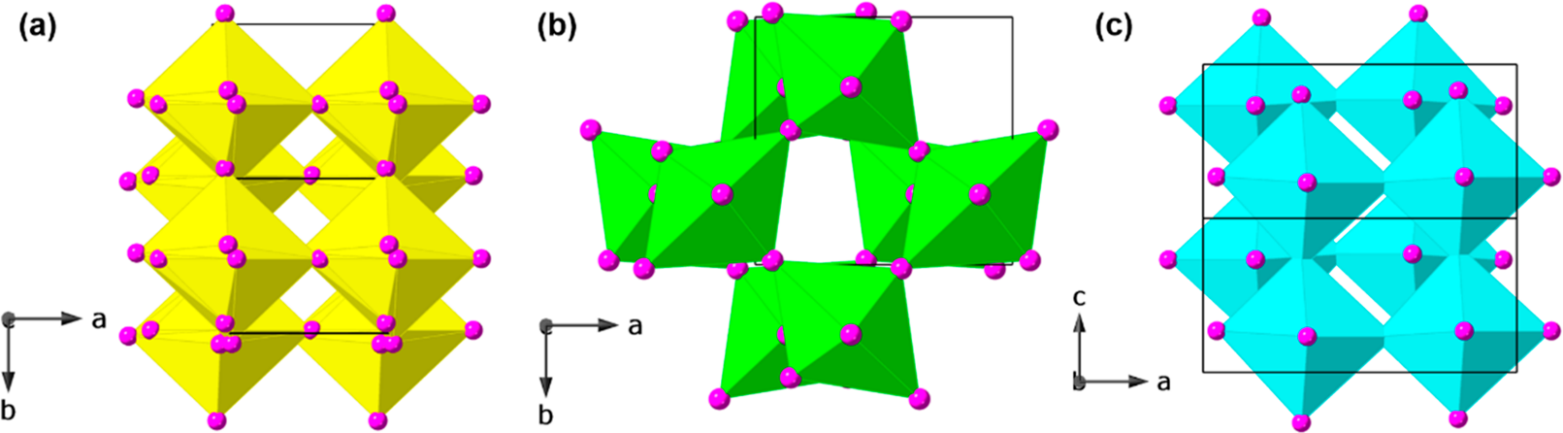
Crystal structures of (a) (2-FbaH)_2_CuCl_4_, (b) (3-FbaH)_2_CuCl_4_, and (c) (4-FbaH)_2_CuCl_4_ at 298 K perpendicular to the layer direction.

In addition to rationalization of unit cell metrics, the space groups of layered perovskites can also often be understood in terms of the underlying types of distortion of the inorganic framework, bearing in mind, of course, that it is ultimately the cooperative relationship of the organic guests and the inorganic framework that dictates these distortions. In general, for layered hybrid perovskites there are two main types of distortion mode, viz. tilting of octahedral MX_6_ units and relative lateral shifts of adjacent [MX_4_]_∞_ layers. In addition, in copper halide perovskites, there is also the J–T mode, which leads to alternating long and short in-plane Cu–Cl bonds. In the case of (3-FbaH)_2_CuCl_4_, use of the online tool ISODISTORT^29^ reveals that the key “standard” distortion modes^7^ are the layer shift mode, together with two distinct octahedral tilt modes (i.e., both rotation around the *c*-axis and out-of-plane tilting, leading to the common Glazer-like tilt system *a*^–^*a*^–^*c*/–(*a*^–^*a*^–^*c*)). The additional significant modes are the J–T mode and a polar mode, acting along the *c*-axis. We note that the [CuCl_4_]_∞_ framework is only marginally affected by the polar mode, which is clearly driven by the positioning of the interlayer moieties; indeed, the space group would be close to centrosymmetric, either *Pbca* or *Pbcm* based on the distortions of the inorganic layer only. Attempts to derive a plausible model in these centrosymmetric space groups proved impossible. An example of the *Pbca* space group (which permits the two octahedral tilt modes, but not the layer shift mode) and corresponding unit cell metrics occurs in (NO_2_C_6_H_4_NH_3_)CuCl_4_.^30^ In the remaining two present examples, matters are complicated by some structural disorder within the [CuCl_4_]_∞_ sheets (Figure S4). After trial refinements, the occupancies of the disordered sites were fixed at 50:50, in each case; whether this disorder is correlated to the retention of centrosymmetricity in these cases is unclear. In the case of (4-FbaH)_2_CuCl_4_, there is no octahedral tilting, but there is a layer shift mode and an additional slight “rippling” of the [CuCl_4_]_∞_ sheets, which leads to the doubled *a*-axis. Such a rippling effect is rare in layered perovskites, but this has been discussed in our recent review^7^ and has been seen in the lead halide (2-fluoroethylammonium)_2_PbBr_4_,^31^ which has the same generic cell metrics and same space group, *Pnma*. A more dramatic example also occurs in a recently reported polymorph of the copper halide [C_6_H_5_(CH_2_)_4_NH_3_]_2_[CuCl_4_].^32^ The additional disorder within the [CuCl_4_]_∞_ layers is effectively a disorder of the J–T affected “long” and “short” in-plane bonds. Such an effect has previously been noted in copper halides, for example, the chiral ferromagnets (*R*-MPEA)_2_CuCl_4_ and (*S*-MPEA)_2_CuCl_4_.^33^ The disorder in (2-FbaH)_2_CuCl_4_ is more complex, and it exhibits aspects of both the J–T disorder and localized, disordered octahedral tilting, similar to that we recently reported in (3-AbaH)_2_CuCl_4_ (3-AbaH^+^ = protonated 3-aminobenzoic acid), which crystallizes in the triclinic space group *P*-1.^34^Figure 2 shows that the “layer shift” degree of freedom for each of the three structure differs significantly: these differences are discussed below in the context of the interactions of the interlayer organic moieties with the [CuCl_4_]_∞_ layers.

The Cu^2+^ ions in halide perovskite structures are strongly J–T active, giving rise to dramatic differences in the Cu–Cl bond lengths and hence to structural distortions of the CuCl_6_ octahedra, with the shorter in-plane bond designated *R*_S_, the longer one *R*_L_, and the out-of-plane bond *R*_Z_ (Table 2). We quantitatively measure the magnitude of the distortion of the octahedra using the equation (2/√3)[*R*_*L*_- (*R*_*S*_ + *R*_*Z*_)/2],^35^ indicating that there is no substantial difference in structural distortion for the three compounds.

**Figure 3:**
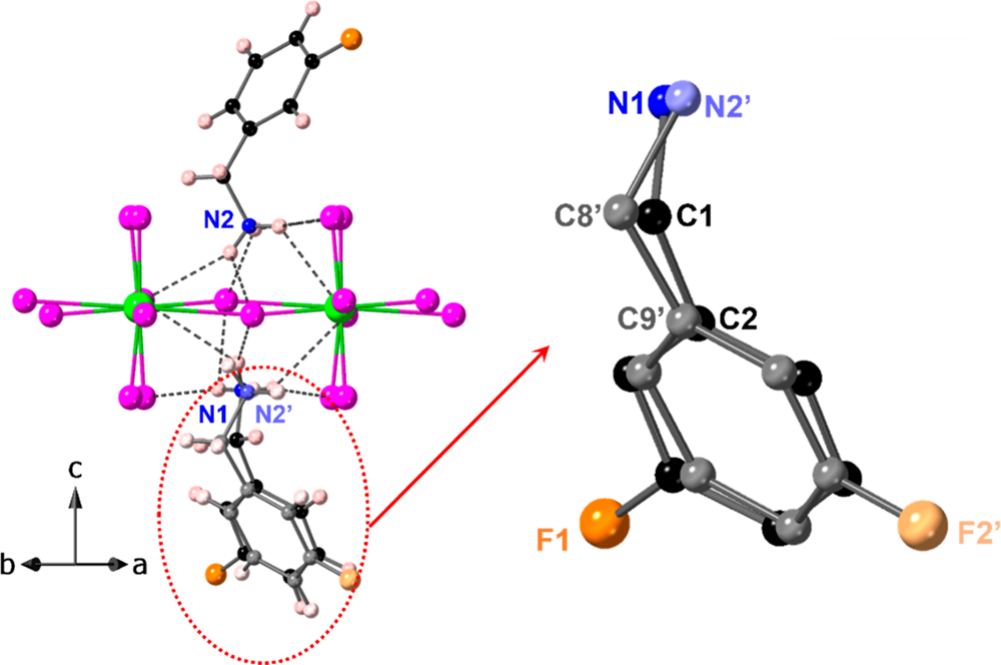
Symmetry breaking in (3-FbaH)_2_CuCl_4_. On the left, the true structure is overlaid with a “dummy” 3-Fba moiety generated by reflection through the Cu–Cl plane. The resulting two overlapping FbaH molecules are shown on the right, which clearly shows one of the key features breaking symmetry to be the positioning of the fluorine atom, in addition to deviations of the −CH_2_NH_3_ group. All hydrogen atoms are omitted for clarity.

**Figure 4:**
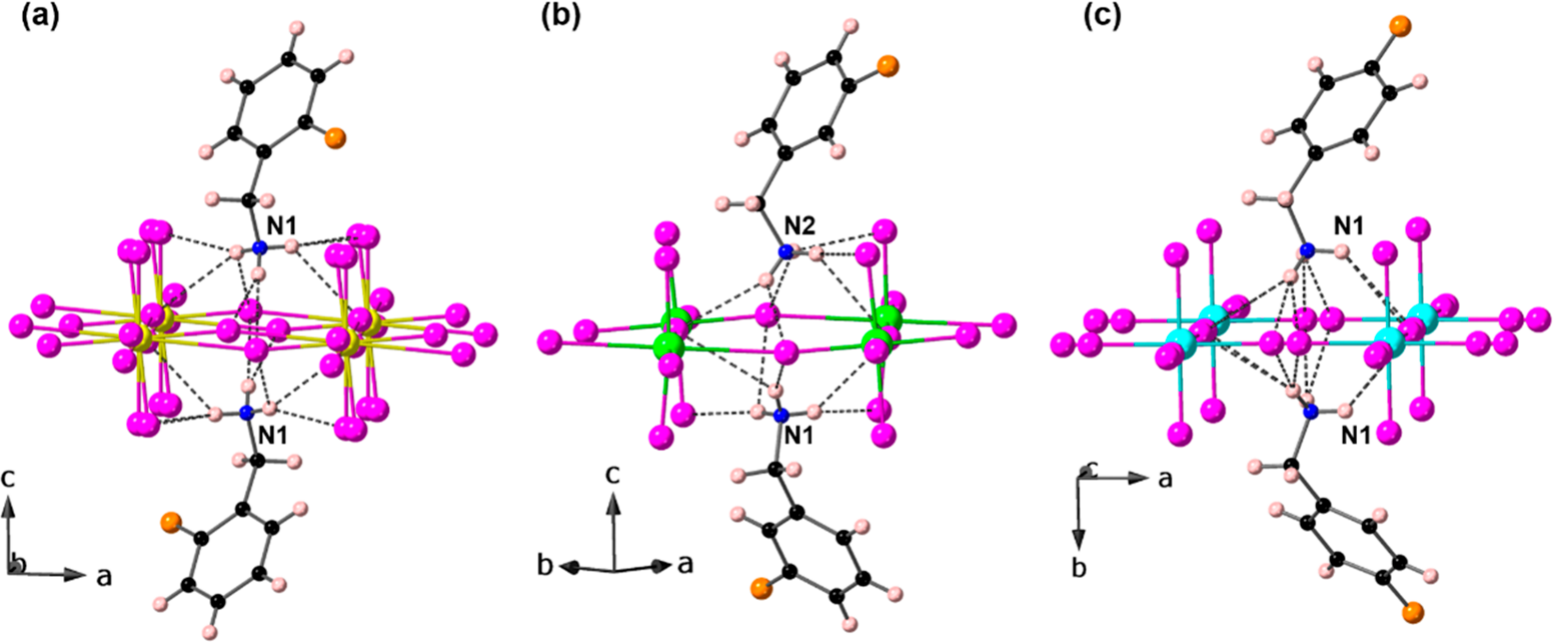
Hydrogen-bonding interactions for (a) (2-FbaH)_2_CuCl_4_, (b) (3-FbaH)_2_CuCl_4_, and (c) (4-FbaH)_2_CuCl_4_ at 298 K. Note the symmetrical arrangements of the FbaH moieties in (a) and (c) and the symmetry breaking in (b). See the Supporting Information for full details of the H-bonding schemes.

At each temperature studied, (3-FbaH)_2_CuCl_4_ crystallizes in the polar orthorhombic system *Pca*2_1_ space group, whereas both (2-FbaH)_2_CuCl_4_ and (4-FbaH)_2_CuCl_4_ crystallize in the centrosymmetric monoclinic *P*2_1_/*c* and orthorhombic *Pnma* space groups, respectively. The protonated fluorobenzylammonium cations in all three structures are ordered and connected with the [CuCl_4_]_∞_ layers through N–H···Cl hydrogen bonds. In the case of (3-FbaH)_2_CuCl_4_ the nature of the lowering of symmetry can be seen in Figure 3, which shows an expanded view of two FbaH moieties and the nature of their H-bonding to a single [CuCl_4_]_∞_ layer. In the corresponding centrosymmetric structure (i.e., that allowing the layer shift mode and the octahedral rotation, but not octahedral tilt, mode, and space group *Pbcm*, discussed above) the Cu atoms would sit on an inversion center, and the two FbaH moieties would be related by a mirror plane through the CuCl_4_ plane (Figure 4). It can be seen that the positioning of the two FbaH moieties breaks this symmetry slightly, with a similar, but distinct, H-bonding network on either side of the plane. In particular, there is a significantly short N–H---Cl contact (H---Cl ∼ 2.38 Å) on one side of the plane, with the corresponding contact on the opposite side of the plane being 2.55 Å. Although the underlying reason for this symmetry breaking is not clear, we might speculate that this is correlated to the ordering of the [CuCl_4_]_∞_ layer itself, which is not seen in either the 2-FbaH or 4-FbaH analogues. Further details of the symmetry-breaking are given in Figure S5 and Table S13.

As shown in Figure 4, in (2-FbaH)_2_CuCl_4_ and (4-FbaH)_2_CuCl_4_ the corresponding interactions of the organic moieties with the [CuCl_4_]_∞_ layer retain their ideal higher symmetry (specifically, the two moieties are related by an inversion center in (2-FbaH)_2_CuCl_4_ and a mirror plane in (4-FbaH)_2_CuCl_4_). However, the (4-FbaH^+^) moieties in the (4-FbaH)_2_CuCl_4_ structure only form N–H···Cl hydrogen bonds with in-plane chlorides, Cl(2) and Cl(3), with no further H-bonding to the apical chloride Cl(1). In each structure, the two FbaH moieties per formula unit form molecular “bilayers” in the interlayer region between the [CuCl_4_]_∞_ layers (Figure 1). In both the (2-FbaH)_2_CuCl_4_ and (4-FbaH)_2_CuCl_4_ structures, the organic amines are arranged with the aromatic groups and F substituents oriented in the middle of these organic bilayers, forming stronger hydrogen bonds, N–H···Cl < 2.78 and 2.83 Å for (2-FbaH)_2_CuCl_4_ and (4-FbaH)_2_CuCl_4_, respectively (Tables S4 and S9). However, the (3-FbaH^+^) moieties in the noncentrosymmetric structure (3-FbaH)_2_CuCl_4_ form a wider range of hydrogen bonds, with weaker contacts up to N–H···Cl = 2.96 Å in addition to the shortest contact of 2.38 Å (Table S6). Thus, the orientation of the cations within each organic bilayer contributes to the changes in crystal symmetry.

F---F intermolecular interactions are known to play a minor, complementary role in determining crystal packing.^36^ In (3-FbaH)_2_CuCl_4_ and (4-FbaH)_2_CuCl_4_, the *intra*-bilayer cohesion may be further enhanced by weak intermolecular F---F interactions (∼3.91and 3.64 Å, respectively) (Figure 1). In contrast, in the (2-FbaH)_2_CuCl_4_ structure, the amines are unable to orient to produce optimized F---F interactions, and the F atoms are inevitably directed toward the [CuCl_4_]_∞_ planes rather than the *intra*-bilayer region. The subtle differences of the overall *inter*-layer cohesion result in different degrees of layer shift of neighboring [CuCl_4_]_∞_ layers in all three structures. Figure 2 shows that neighboring [CuCl_4_]_∞_ layers in both the (3-FbaH)_2_CuCl_4_ and (4-FbaH)_2_CuCl_4_ structures are staggered relative to each other in a style that spans the spectrum from DJ-like to RP-like. Both structures appear to be near RP, with a greater degree of layer shift in the (4-FbaH)_2_CuCl_4_ structure, consistent with the enhanced intermolecular F---F interaction (∼3.64 Å). In contrast, in the (2-FbaH)_2_CuCl_4_ structure, the adjacent octahedral layers are partially eclipsed, in the DJ2 style, despite the stoichiometry.

The positional isomerism of closely related amines clearly has a significant effect on the structural behavior observed. A similar effect has been observed in corresponding lead chloride systems.^37^ However, the structural details and by implication the underlying factors driving polar behavior in only one of the isomers are subtly different. In the present systems, introducing fluorine at the *ortho*- and *para*-positions retains centrosymmetric symmetry, whereas via *meta*-substitution the crystal symmetry is reduced to noncentrosymmetric. In the case of the lead chloride analogues, it is the (2-FbaH)_2_PbCl_4_ isomer that is polar (and ferroelectric), whereas the *meta* and *para* isomers are centrosymmetric. Furthermore, the polar axis in (2-FbaH)_2_PbCl_4_ lies along an in-plane direction, whereas in (3-FbaH)_2_CuCl_4_ it lies *perpendicular* to the [CuCl_4_]_∞_ layers (i.e., along the *c*-axis). The reasons for this are presumably dictated by the subtle differences in H-bonding opportunities, but identifying an exact comparison is difficult due to the two unique features of the Cu-based systems, i.e., the J–T effect and the disordering within the [CuCl_4_]_∞_ layers, neither of which is present in the Pb-based systems. A potential lone pair effect from Pb^2+^ may also contribute toward the observed differences in structure.

### Electrical Properties of (3-FbaH)_2_CuCl_4_

The relative permittivity measured of the polar compound (3-FbaH)_2_CuCl_4_ at 100 kHz appears to show a peak at ca. 426 K on both heating and cooling (Figure S6). However, no peak was observed in data obtained at several other frequencies and instead showed a large dispersion with permittivity values in excess of 10000 at 1 kHz and below; this indicated that the peak observed at 100 kHz is not intrinsic but is instead linked to the increasing influence of electrode polarization and changing contact resistance.^38−40^ Collection of dielectric data at higher temperature was not possible as the sample decomposed. Variable temperature PXRD was also performed to check for the presence of a structural transition, possibly associated with a ferroelectric *T*_C_. On heating from 298 to 413 K the diffraction patterns remain unchanged, but the data at 433 K is notably different with the appearance of new diffraction peaks. The single-crystal structure of (3-FbaH)_2_CuCl_4_ above 400 K has not been determined because the diffraction in the high-temperature phase is too weak. Despite the polar nature of (3-FbaH)_2_CuCl_4_, the combination of dielectric and diffraction data does not support the presence of a ferroelectric-to-paraelectric transition in the temperature range investigated. It may be that the compound decomposes before this transition.

**Figure 5:**
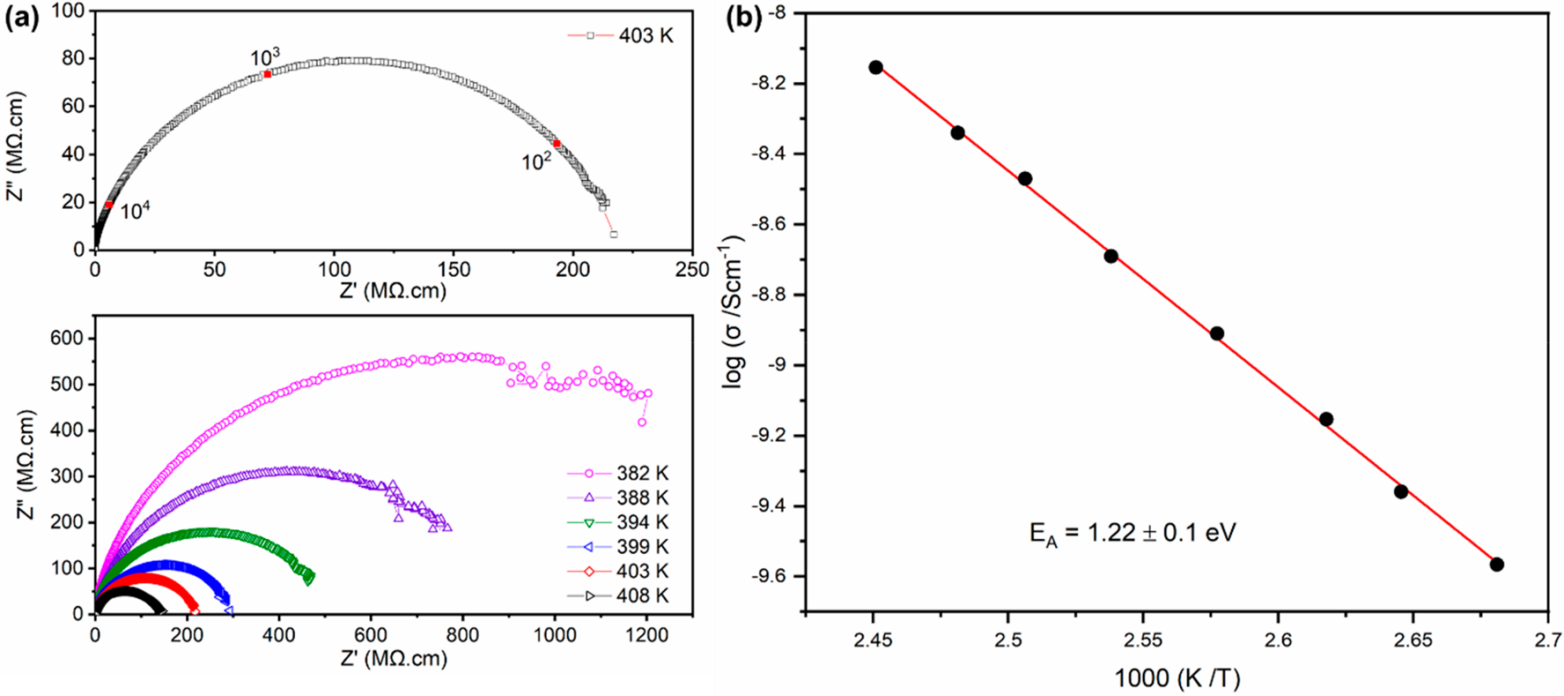
(a) Complex impedance plane (Z*) plots for (3-FbaH)_2_CuCl_4_ as a function of temperature showing a single semicircular arc associated with a bulk response. (b) Arrhenius plot of bulk conductivity as a function of temperature (*R*^2^ = 0.9993).

The electrical properties of (3-FbaH)_2_CuCl_4_ were also investigated by using impedance spectroscopy measurements from room temperature up to 408 K. Above 382 K, the complex impedance, Z*, response indicated a single semicircular arc which decreased in size with increasing temperature (Figure 5a). This indicates a decrease in resistivity with increasing temperature and semiconducting behavior. The semicircular arc was fitted assuming a single parallel resistor–capacitor (R–C) circuit and had an associated capacitance of 1–2 pF at all temperatures and is consistent with a bulk response.^41^ Bulk resistivity (ρ) values were estimated from the intercepts of the semicircular arc with the *x*-axis (the real part of the complex impedance, *Z*′) and converted to bulk conductivity, σ = 1/ρ, and plotted in Arrhenius format (Figure 5b). The data show linear behavior consistent with thermally activated semiconducting properties and with an activation energy of 1.22 ± 0.1 eV. Unfortunately, the level of conductivity means that polarization switching was not possible to conclusively demonstrate ferroelectricity.

### Magnetic Properties

**Figure 6:**
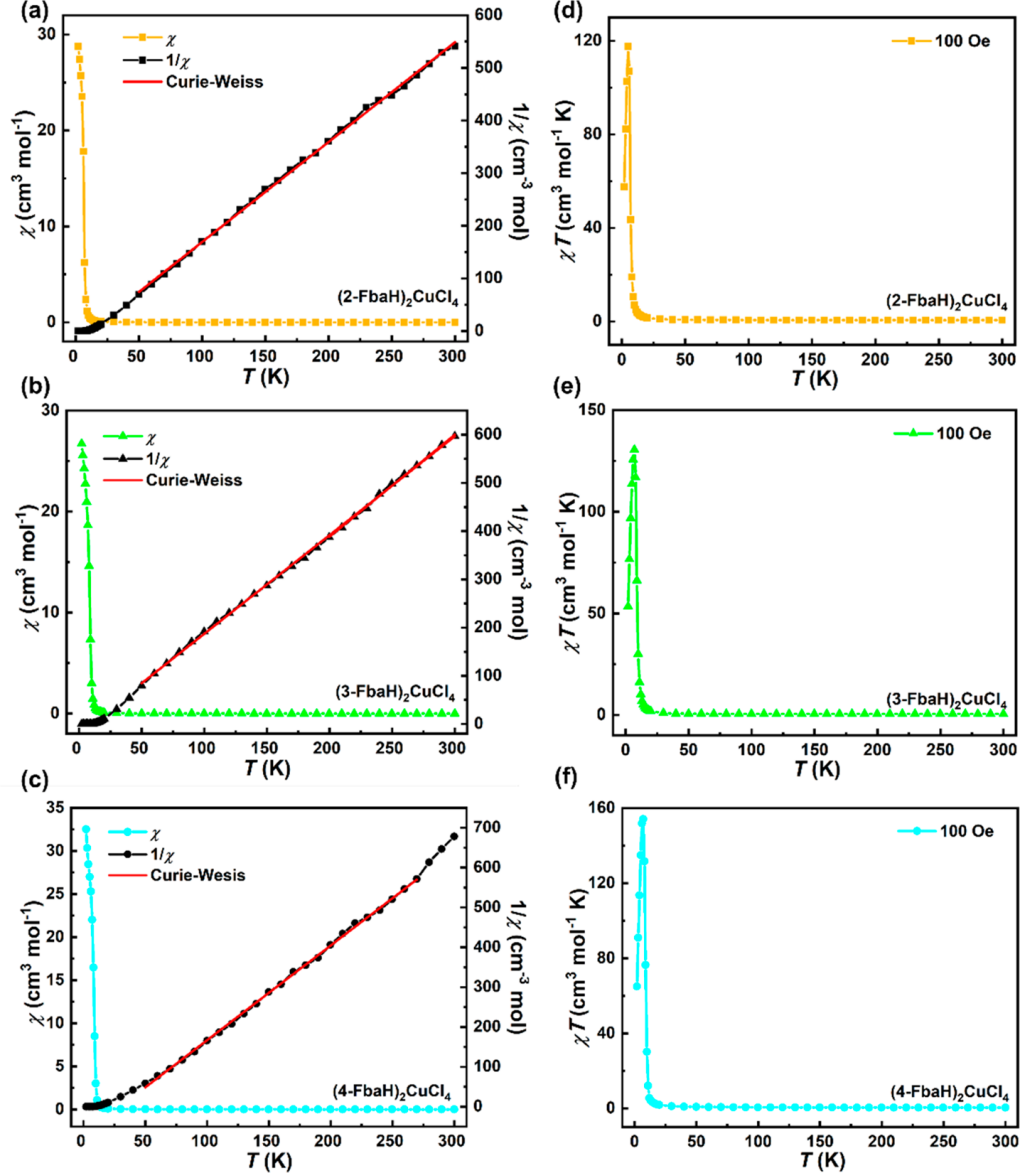
Magnetic susceptibility (χ) and its inverse 1/χ with Curie–Weiss fit (red line) for (a) (2-FbaH)_2_CuCl_4_ in the region 50–300 K, (b) (3-FbaH)_2_CuCl_4_ in the region 50–300 K, and (c) (4-FbaH)_2_CuCl_4_ in the region 50–270 K. χ*T* vs *T* for (d) (2-FbaH)_2_CuCl_4_, (e) (3-FbaH)_2_CuCl_4_, and (f) (4-FbaH)_2_CuCl_4_.

The magnetic measurements have been performed in the temperature region 2–300 K with an applied dc field of 100 Oe. Figures 6a–c show the magnetic susceptibility (χ) measured as a function of decreasing temperature *T* in an applied magnetic field, where χ increases gradually with decreasing *T* and then increases very rapidly below around 10 K. No maximum is observed in the χ vs *T* plots, consistent with the spontaneous onset of ferromagnetic order. The dominance of ferromagnetic interactions within the [CuCl_4_]_∞_ layers resembles previously reported 2D layered copper(II) perovskites^42−44^ and is further reflected in the high-temperature magnetic response. The inverse susceptibility (1/χ) data were fitted to the Curie–Weiss law in the region 50–300 K for (2-FbaH)_2_CuCl_4_ and (3-FbaH)_2_CuCl_4_ and 50–270 K for (4-FbaH)_2_CuCl_4_. This gave the fitted values: Curie constant, *C* = 0.53(5), 0.50(2), and 0.46(3) cm^3^ mol^–1^ K, Weiss constant θ = 10.8(2), 8.8(1), and 32.2(3) K, derived effective moment, μ_eff_ = 2.059(5), 2.000(2), and 1.918(3) μ_B_, and Landé factor, *g* = 2.378, 2.309, and 2.215 for (2-FbaH)_2_CuCl_4_, (3-FbaH)_2_CuCl_4_, and (4-FbaH)_2_CuCl_4_, respectively, which are within the expected range for one Cu^2+^ ion with *S* = 1/2.^42,43^ The peaks in χ*T* vs *T* plots (Figure 6d–f), occurring at 5 K for (2-FbaH)_2_CuCl_4_ and 7 K for (3-FbaH)_2_CuCl_4_ and (4-FbaH)_2_CuCl_4_, simply reflect the approach to saturation of the ordered ferromagnetic moment as the temperature is lowered, which is further reinforced by the field-dependent magnetic response discussed below. The polar ferromagnet (3-FbaH)_2_CuCl_4_ therefore exhibits the potential for multiferroic behavior.

**Figure 7:**
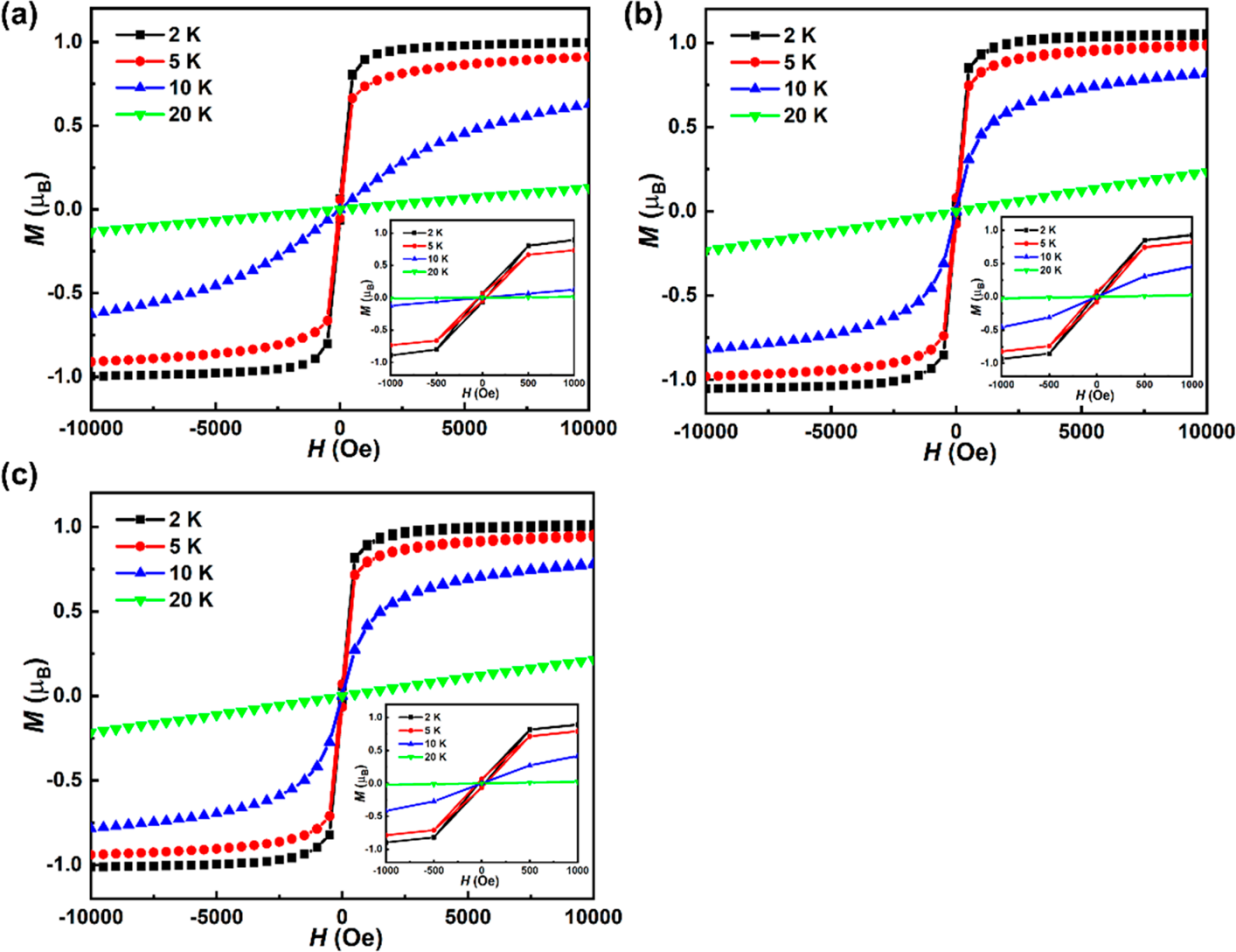
Magnetization (*M*) vs magnetic field (*H*) at 2, 5, 10, and 20 K for (a) (2-FbaH)_2_CuCl_4_, (b) (3-FbaH)_2_CuCl_4_, and (c) (4-FbaH)_2_CuCl_4_, respectively. Inset: the low-field region of the hysteresis loops.

The magnetization vs magnetic field plots for these three compounds at 2, 5, 10, and 20 K are shown in Figure 7. Below the ordering temperatures of around 10 K all three compounds exhibit sharp magnetic switching at low field with a small amount of hysteresis, in contrast to the linear paramagnetic response at 20 K. All compounds display hysteresis loops at 2 and 5 K, with a coercive field of approximately 40 Oe for (2-FbaH)_2_CuCl_4_, 25 Oe for (3-FbaH)_2_CuCl_4_, and (4-FbaH)_2_CuCl_4_ at 2 K (Figure S9). At 2 K, the magnetic moment at low field has saturation values of approximately 1.00, 1.03, and 1.01 μ_B_ for (2-FbaH)_2_CuCl_4_, (3-FbaH)_2_CuCl_4_, and (4-FbaH)_2_CuCl_4_, respectively, which are comparable to the expected value (1.0 μ_B_) for an *S* = 1/2 system.^42−44^ Furthermore, the hysteresis effect observed in all compounds is characteristic of a soft ferromagnet.

The most reliable estimate of the ground state comes from saturation magnetization below *T*_c_, which is in excellent agreement with that expected value of *M*_s_ = 1 μ_B_ for an *S* = 1/2 spin only ground state. The values of effective moment derived at higher temperature in the paramagnetic state are about 10% higher than the expected value of 1.73*M*_s_ under the assumption of no additional contributions to the moment at higher temperature (i.e., constant Curie constant). The existence of ferromagnetic correlations in the [CuCl_4_]_∞_ layers can be understood as arising from the motif of alternating in-plane long and short bonds at neighboring Cu sites such that the superexchange along the nearly linear Cu–Cl–Cu path is controlled by the Hund’s rule correlations at the ligand. As here, the development of long-range ferromagnetic order is also observed in other 2D layered copper(II) perovskites,^42−44^ which due to the Mermin–Wagner theorem demands the existence some additional source of anisotropy. Recent DFT calculations on related [CuCl_4_]_∞_-based systems suggest this may be a single ion anisotropy arising from spin–orbit interactions due to the large covalency between the Cu and the Cl.^45^ The resulting additional moment, estimated as ∼0.1 μ_B_, may also help account for the higher effective moment observed in the paramagnetic state in our materials.

## Conclusions

In summary, we have explored the effect of structural isomerism of the interlayer organic cation in the series of layered perovskites (*n*-FbaH)_2_CuCl_4_ (*n* = 2, 3, 4). Fluorination of benzylamine at the *ortho*- and *para*-positions leads to layered perovskites (2-FbaH)_2_CuCl_4_ and (4-FbaH)_2_CuCl_4_ which retain centrosymmetric structures. However, the effects of resulting interactions with the inorganic framework, and also of differing weaker interactions within the organic bilayers, lead to differences in relative shifts of the [CuCl_4_]_∞_ layers within each of the compounds and also different octahedral tilting arrangements, with both these compounds exhibiting disordering of Cl ligands. More significantly, *meta-*F-substitution in (3-FbaH)_2_CuCl_4_ leads to a polar material, with the symmetry being broken by subtle displacements in the positions of the 3-FbaH moieties, with resulting advantages in H-bonding opportunities to the [CuCl_4_]_∞_ layers. This symmetry-breaking mechanism seems related to, but distinct from, that recently seen in some manganese chloride layered perovskites^46^ where the rotational degree of freedom of extra-cyclic chains is suggested to be responsible. In addition, in this case, we see that the F-substitution of the phenyl ring also plays a key role. All three compounds display ferromagnetic interactions, and therefore polar (3-FbaH)_2_CuCl_4_ may be a type I multiferroic, although ferroelectric switching has yet to be demonstrated. It is notable that there are no phase transitions in any of the three present compounds in the range 93–293 K. In each case the organic moieties are well-ordered throughout this regime, but in (2-FbaH)_2_CuCl_4_ and (4-FbaH)_2_CuCl_4_ the inorganic layers remain disordered. It might be anticipated that a more ordered ground state would freeze out on further cooling, and perhaps this would ultimately lead to a polar phase for these compositions at temperatures below 93 K. The non-fluorinated parent structure (BaH)_2_CuCl_4_ also exhibits disordered inorganic [CuCl_4_]_∞_ layers and a centrosymmetric space group. From this we might speculate that there is a cooperativity between ordering of the [CuCl_4_]_∞_ layers and ordering of the interlayer organic moieties into a stable noncentrosymmetric disposition, a phenomenon which is worthy of further study.

## Supporting Information Available

Rietveld refinements of PXRD data, crystallographic data at 93 and 173 K, thermogravimetric analysis data, hydrogen-bonding details, the details of symmetry breaking of (3-FbaH)_2_CuCl_4_, dielectric permittivity and variable-temperature PXRD data of (3-FbaH)_2_CuCl_4_, magnetic data of three compounds (PDF)

## Accession Codes

CCDC 2122978–2122985 contain the supplementary crystallographic data for this paper. These data can be obtained free of charge via www.ccdc.cam.ac.uk/data_request/cif, or by emailing data_request@ccdc.cam.ac.uk, or by contacting The Cambridge Crystallographic Data Centre, 12 Union Road, Cambridge CB2 1EZ, UK; fax: +44 1223 336033.

## Notes

The research data supporting this publication can be accessed at https://doi.org/10.17630/fed6da3b-6efe-46e9-8ed0-488f6e43a4b8.
