# Supplementary material for: Polar Ferromagnet Induced by Fluorine Positioning in Isomeric Layered Copper Halide Perovskites
Source: Inorg Chem. 2022 Feb 9;61(7):3230–9. doi: 10.1021/acs.inorgchem.1c03726 (PMC9007457; doi:10.1021/acs.inorgchem.1c03726)
Supplement: Supplementary file 1 — ic1c03726_si_001.pdf [file ic1c03726_si_001.pdf]

Polar ferromagnet induced by fluorine positioning in isomeric layered copper halide perovskites

Ceng Han,[†] Jason A. McNulty,[†] Alasdair J. Bradford,[†,‡] Alexandra M. Z. Slawin,[†] Finlay D. Morrison,[†] Stephen L. Lee,[‡] and Philip Lightfoot*,[†]

[†]School of Chemistry and EaStChem, University of St Andrews, St Andrews, KY16 9ST, UK

[‡]School of Physics, University of St Andrews, St Andrews, Fife, KY16 9SS, UK

*e-mail: pl@st-andrews.ac.uk

Supplementary Information

Figure S1. The full-range (left) and expanded (right) PXRD data for (a) $(2\text{-FbaH})_2CuCl_4$, (b) $(3\text{-FbaH})_2CuCl_4$ and (c) $(4\text{-FbaH})_2CuCl_4$.

Figure S2. Full-range and expanded (inset) Rietveld plot (PXRD) for (a) $(2\text{-FbaH})_2CuCl_4$, (b) $(3\text{-FbaH})_2CuCl_4$ and (c) $(4\text{-FbaH})_2CuCl_4$. Note that there is a significant preferred orientation correction applied. In addition, only unit cell and profile parameters were refined, not atomic parameters. The aim here is merely to demonstrate phase purity.

Figure S3. Thermogravimetric analysis (TGA) data for (a) $(2\text{-FbaH})_2CuCl_4$, (b) $(3\text{-FbaH})_2CuCl_4$ and (c) $(4\text{-FbaH})_2CuCl_4$.

Figure S4. The nature of the distortion within the $CuCl_6$ octahedra in (a) $(2\text{-FbaH})_2CuCl_4$, (b) $(3\text{-FbaH})_2CuCl_4$, and (c) $(4\text{-FbaH})_2CuCl_4$ at 298 K.

Figure S5. The symmetry breaking of $(3\text{-FbaH})_2CuCl_4$. The true structure is shown on the left in (a). The middle picture (b) shows a simulated representation of the second molecule derived by application of mirror symmetry through the Cu-Cl plane. The right-hand image (c) overlays the real structure with the structure derived in (b).

Figure S6. (a) Real part ε_r of the dielectric permittivity for $(3\text{-FbaH})_2CuCl_4$ at 100 KHz in heating and cooling runs. (b) Variable-temperature PXRD patterns from room temperature to 433 K.

Figure S7. Thermal evolution of the lattice metrics obtained from Rietveld refinement of SXRD data for $(3\text{-FbaH})_2CuCl_4$ at the range 290 K to 440 K.

Figure S8. ZFC and FC curves at 100 Oe from 2 to 20 K for (a) $(2\text{-FbaH})_2CuCl_4$, (b) (3-

FbaH)$_2$CuCl$_4$ and (c) (4-FbaH)$_2$CuCl$_4$.

Figure S9. Magnetization (*M*) versus magnetic field (*H*) at 2, 5, 10 and 20 K between -500 to 500 Oe for (a) (2-FbaH)$_2$CuCl$_4$, (b) (3-FbaH)$_2$CuCl$_4$ and (c) (4-FbaH)$_2$CuCl$_4$. Inset: the low-field region -100 to 100 Oe of the hysteresis loops at 2 K.

Table S1. Crystallographic data and refinement details for (2-FbaH)$_2$CuCl$_4$, (3-FbaH)$_2$CuCl$_4$ and (4-FbaH)$_2$CuCl$_4$ at 93 and 173 K.

Table S2. Hydrogen bond lengths (Å) and angles (°) for (2-FbaH)$_2$CuCl$_4$ at 93 K.

Table S3. Hydrogen bond lengths (Å) and angles (°) for (2-FbaH)$_2$CuCl$_4$ at 173 K.

Table S4. Hydrogen bond lengths (Å) and angles (°) for (2-FbaH)$_2$CuCl$_4$ at 298 K.

Table S5. Hydrogen bond lengths (Å) and angles (°) for (3-FbaH)$_2$CuCl$_4$ at 173 K.

Table S6. Hydrogen bond lengths (Å) and angles (°) for (3-FbaH)$_2$CuCl$_4$ at 298 K.

Table S7. Hydrogen bond lengths (Å) and angles (°) for (4-FbaH)$_2$CuCl$_4$ at 93 K.

Table S8. Hydrogen bond lengths (Å) and angles (°) for (4-FbaH)$_2$CuCl$_4$ at 173 K.

Table S9. Hydrogen bond lengths (Å) and angles (°) for (4-FbaH)$_2$CuCl$_4$ at 298 K.

Table S10. Atomic coordinates of (2-FbaH)$_2$CuCl$_4$ at 298 K.

Table S11. Atomic coordinates of (3-FbaH)$_2$CuCl$_4$ at 298 K.

Table S12. Atomic coordinates of (4-FbaH)$_2$CuCl$_4$ at 298 K.

Table S13. Three-dimensional distances of the two overlapping (3-FbaH$^+$) molecules.

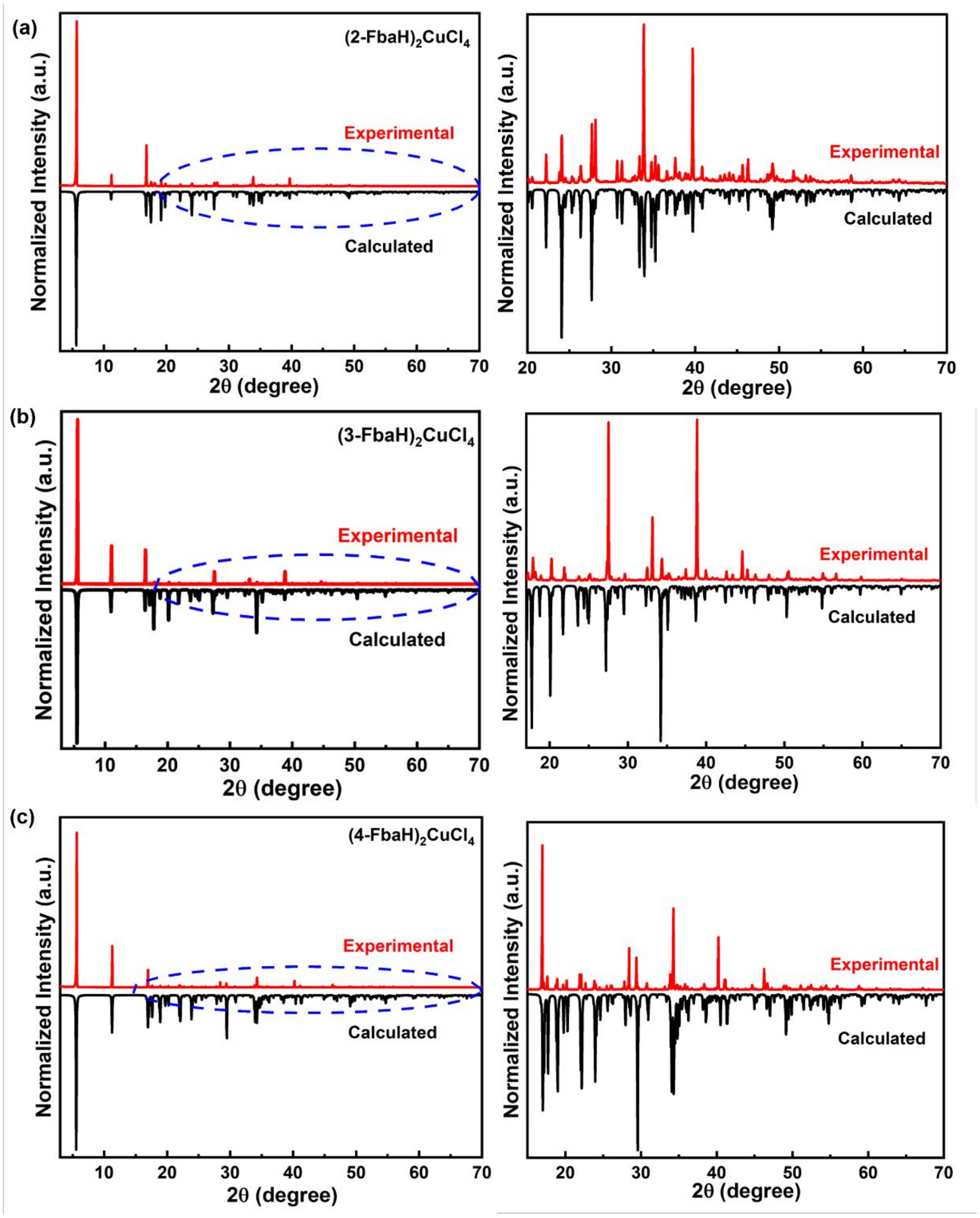

Figure S1. The full-range (left) and expanded (right) PXRD data for (a) $(2\text{-FbaH})_2CuCl_4$, (b) $(3\text{-FbaH})_2CuCl_4$ and (c) $(4\text{-FbaH})_2CuCl_4$.

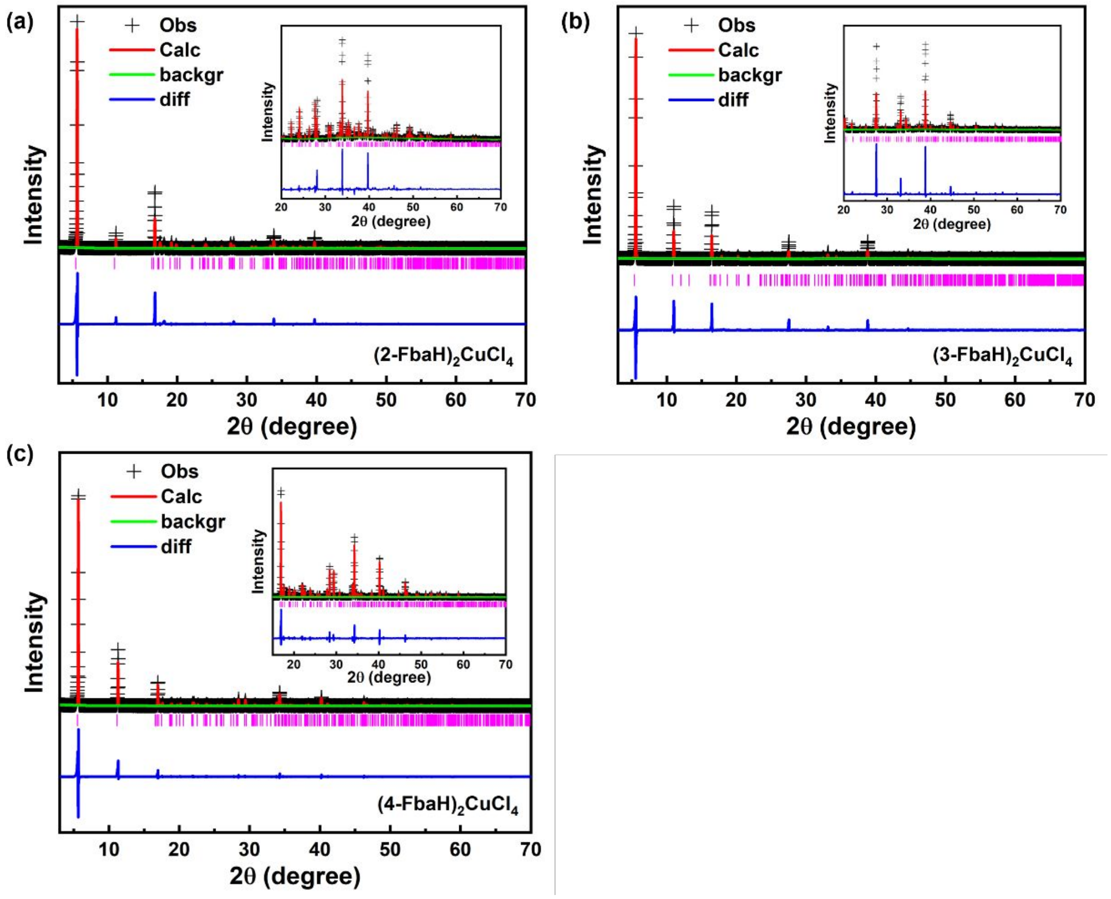

Figure S2. Full-range and expanded (inset) Rietveld plot (PXRD) for (a) $(2\text{-FbaH})_2CuCl_4$, (b) $(3\text{-FbaH})_2CuCl_4$ and (c) $(4\text{-FbaH})_2CuCl_4$. Note that there is a significant preferred orientation correction applied. In addition, only unit cell and profile parameters were refined, not atomic parameters. The aim here is merely to demonstrate phase purity.

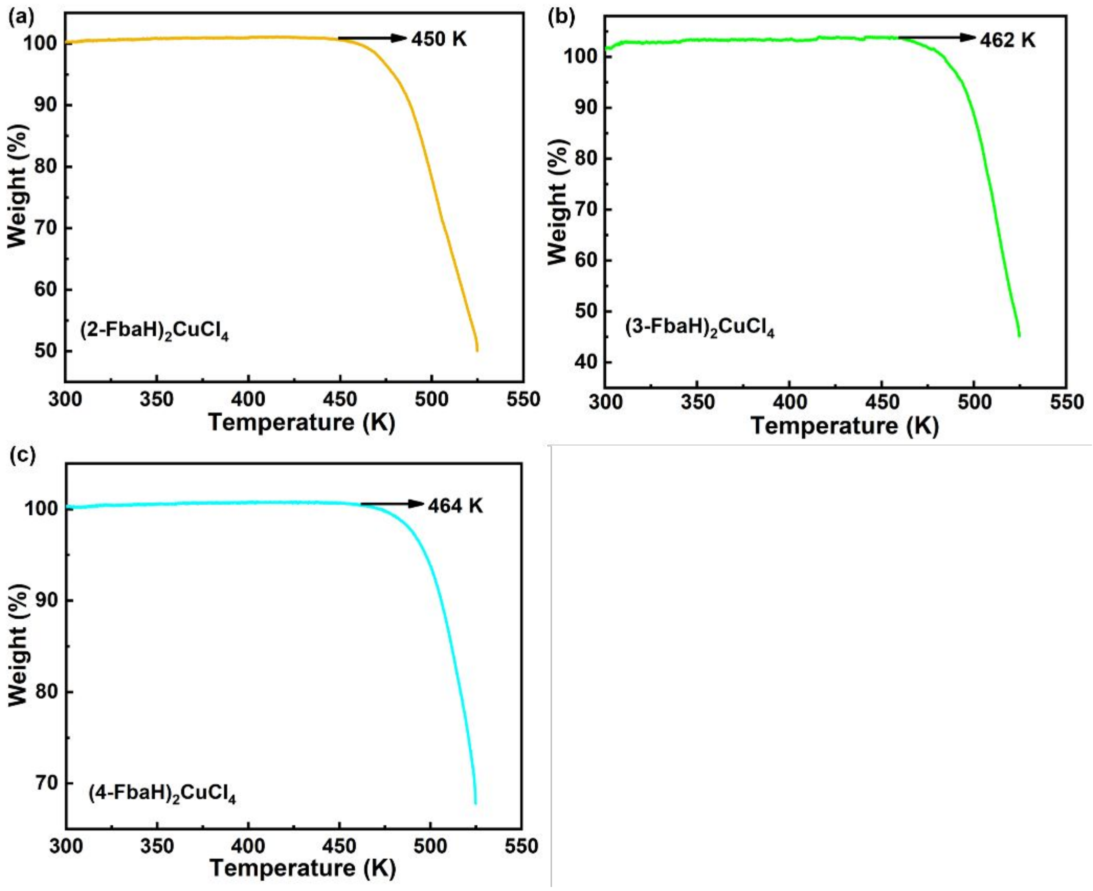

Figure S3. Thermogravimetric analysis (TGA) data for (a) (2-FbaH)$_2$CuCl$_4$, (b) (3-FbaH)$_2$CuCl$_4$ and (c) (4-FbaH)$_2$CuCl$_4$.

Table S1. Crystallographic data and refinement details for $(2\text{-FbaH})_2CuCl_4$, $(3\text{-FbaH})_2CuCl_4$ and $(4\text{-FbaH})_2CuCl_4$ at 93 and 173 K.

compound	$(2\text{-FbaH})_2CuCl_4$	$(2\text{-FbaH})_2CuCl_4$	$(3\text{-FbaH})_2CuCl_4$	$(4\text{-FbaH})_2CuCl_4$	$(4\text{-FbaH})_2CuCl_4$
**temperature (K)**	93	173	173	93	173
**formula**	$C_{14}H_{18}F_2N_2CuCl_4$	$C_{14}H_{18}F_2N_2CuCl_4$	$C_{14}H_{18}F_2N_2CuCl_4$	$C_{14}H_{18}F_2N_2CuCl_4$	$C_{14}H_{18}F_2N_2CuCl_4$
**formula weight**	457.64	457.64	457.64	457.64	457.64
**colour/habit**	Green/Platelet	Yellow/Platelet	Yellow/Platelet	Yellow/Platelet	Yellow/Platelet
**crystal size (mm^3)**	0.53 × 0.44 × 0.10	0.25 × 0.25 × 0.08	0.33 × 0.32 × 0.15	0.10 × 0.08× 0.08	0.17 × 0.10 × 0.06
**crystal system**	Monoclinic	Monoclinic	Orthorhombic	Orthorhombic	Orthorhombic
**space group**	$P2_1/c$	$P2_1/c$	$Pca2_1$	*Pnma*	*Pnma*
a **(Å)**	5.3763(4)	5.3750(4)	7.5330(5)	10.581(2)	10.5488(8)
b **(Å)**	5.1258(3)	5.1230(3)	7.2519(5)	31.058(7)	31.1420(2)
c **(Å)**	31.569(2)	31.575(2)	32.298(2)	5.2330(10)	5.2174(4)
α **(deg)**	90	90	90	90	90
β **(deg)**	90.565(3)	90.610(2)	90	90	90
γ **(deg)**	90	90	90	90	90
V **($Å^3$)**	869.93(10)	869.40 (10)	1764.4(2)	1719.7(6)	1713.97(19)
Z	2	2	4	2	4
**ρ_{calc} (mg/m^3)**	1.747	1.748	1.723	1.768	1.774
**μ (mm^{-1})**	1.887	1.888	1.860	1.909	1.915
**F(000)**	462	462	924	924	924
**No. of reflns collected**	7639	6684	15654	10550	15108
**independent reflns**	1937 [R(int) = 0.0402]	1904 [R(int) = 0.0348]	3930 [R(int) = 0.0392]	1526 [R(int) = 0.0450]	1997 [R(int) = 0.0504]
**goodness of fit**	1.215	1.062	1.108	1.060	1.057
**final *R* indices ($I > 2\sigma(I)$)**	R_1 = 0.0768 wR_2 = 0.1828	R_1 = 0.0297 wR_2 = 0.0724	R_1 = 0.0223 wR_2 = 0.0556	R_1 = 0.0329 wR_2 = 0.0823	R_1 = 0.0282 wR_2 = 0.0618
**largest diff. peak/hole (e $Å^{-3}$)**	1.028/-0.561	0.464/-0.322	0.255/-0.270	0.386/-0.527	0.327/-0.362

Table S2. Hydrogen bond lengths (Å) and angles (°) for $(2\text{-FbaH})_2CuCl_4$ at 93 K.

D-H…A	d(D-H)	d(H…A)	d(D…A)	∠(DHA)
N(1)-H(1A)...Cl(1)#1	0.89	2.50	3.323(8)	153.8
N(1)-H(1A)...Cl(1A)#1	0.89	2.66	3.418(8)	143.8
N(1)-H(1A)...Cl(2A)#1	0.89	2.88	3.517(7)	129.5
N(1)-H(1A)...Cl(2A)#2	0.89	2.55	3.202(7)	130.6
N(1)-H(1B)...Cl(2)#3	0.89	2.82	3.627(8)	152.1
N(1)-H(1B)...Cl(2)#4	0.89	2.38	3.225(7)	158.5
N(1)-H(1B)...Cl(2A)#4	0.89	2.70	3.235(7)	120.2
N(1)-H(1C)...Cl(1)#5	0.89	2.58	3.440(9)	164.0
N(1)-H(1C)...Cl(2)#6	0.89	2.73	3.229(7)	116.4

Symmetry transformations used to generate equivalent atoms:

#1 x,y-3,z; #2 -x+2,-y+2,-z; #3 x,y-2,z; #4 -x+3,-y+2,-z; #5 x+1,y-3,z; #6 -x+3,-y+1,-z

Table S3. Hydrogen bond lengths (Å) and angles (°) for $(2\text{-FbaH})_2CuCl_4$ at 173 K.

D-H…A	d(D-H)	d(H…A)	d(D…A)	∠(DHA)
N(1)-H(1A)...Cl(1)#4	0.91	2.40	3.291(2)	164.7
N(1)-H(1A)...Cl(1A)#5	0.91	2.58	3.412(2)	152.4
N(1)-H(1A)...Cl(2)#1	0.91	2.66	3.195(2)	118.2
N(1)-H(1B)...Cl(2A)	0.91	2.74	3.637(2)	171.0
N(1)-H(1B)...Cl(2A)#3	0.91	2.35	3.225(2)	162.6
N(1)-H(1C)...Cl(1)#6	0.91	2.61	3.461(3)	161.4
N(1)-H(1C)...Cl(2)#3	0.91	2.60	3.240(2)	112.3
N(1)-H(1C)...Cl(2A)#7	0.91	2.78	3.215(2)	125.0

Symmetry transformations used to generate equivalent atoms:

#1 -x-4, -y+2, -z; #2 -x-4, -y+3, -z; #3 -x-3,-y+2,-z; #4 x, y-1, z; #5 -x-4, -y+1, -z; #6 x+1, y-1, z; #7 -x-3, -y+1, -z

Table S4. Hydrogen bond lengths (Å) and angles (°) for $(2\text{-FbaH})_2CuCl_4$ at 298 K.

D-H…A	d(D-H)	d(H…A)	d(D…A)	∠(DHA)
N(1)-H(1A)...Cl(1A)#4	0.89	2.59	3.426(3)	156.2
N(1)-H(1A)...Cl(1)#4	0.89	2.44	3.317(3)	168.1
N(1)-H(1A)...Cl(2)	0.89	2.75	3.226(3)	115.0
N(1)-H(1B)...Cl(2A)#5	0.89	2.41	3.259(3)	160.7
N(1)-H(1B)...Cl(2A)#1	0.89	2.78	3.668(3)	174.1
N(1)-H(1C)...Cl(1)#6	0.89	2.63	3.483(3)	160.5
N(1)-H(1C)...Cl(2A)#6	0.89	2.64	3.232(3)	124.9
N(1)-H(1C)...Cl(2)#5	0.89	2.77	3.263(3)	116.0

Symmetry transformations used to generate equivalent atoms:

#1 -x+2, -y+4, -z+1; #2 -x+2, -y+3, -z+1; #3 -x+1, -y+4, -z+1; #4 x, y-1, z; #5 x+1, y, z; #6 x+1, y-1, z

Table S5. Hydrogen bond lengths (Å) and angles (°) for $(3\text{-FbaH})_2CuCl_4$ at 173 K.

D-H…A	d(D-H)	d(H…A)	d(D…A)	∠(DHA)
N(1)-H(1A)...Cl(1)	0.91	2.33	3.212(3)	163.9
N(1)-H(1A)...Cl(2)	0.91	2.87	3.335(4)	113.3
N(1)-H(1B)...Cl(2)#1	0.91	2.85	3.506(4)	130.3
N(1)-H(1B)...Cl(3)#2	0.91	2.55	3.348(3)	146.7
N(1)-H(1C)...Cl(1)#3	0.91	2.70	3.471(4)	142.9
N(1)-H(1C)...Cl(3)#3	0.91	2.58	3.293(3)	135.4
N(2)-H(2A)...Cl(4)	0.91	2.42	3.278(3)	158.2
N(2)-H(2B)...Cl(2)#4	0.91	2.54	3.379(4)	152.7
N(2)-H(2B)...Cl(3)	0.91	2.80	3.368(4)	121.9
N(2)-H(2C)...Cl(2)#5	0.91	2.67	3.325(3)	129.2
N(2)-H(2C)...Cl(4)#6	0.91	2.67	3.415(4)	139.8
N(1)-H(1A)...Cl(1)	0.91	2.33	3.212(3)	163.9

Symmetry transformations used to generate equivalent atoms:
#1 x-1/2, -y+1, z; #2 x, y+1, z; #3 x+1/2, -y+1, z; #4 x-1/2, -y, z; #5 x, y-1, z; #6 x+1/2, -y, z

Table S6. Hydrogen bond lengths (Å) and angles (°) for $(3\text{-FbaH})_2CuCl_4$ at 298 K.

D-H…A	d(D-H)	d(H…A)	d(D…A)	∠(DHA)
N(1)-H(1A)...Cl(1)#1	0.89	2.69	3.473(4)	147.8
N(1)-H(1A)...Cl(3)#1	0.89	2.67	3.326(4)	130.9
N(1)-H(1B)...Cl(2)#2	0.89	2.93	3.521(4)	125.3
N(1)-H(1B)...Cl(3)#3	0.89	2.56	3.369(4)	151.6
N(1)-H(1C)...Cl(1)	0.89	2.38	3.256(3)	166.7
N(1)-H(1C)...Cl(2)	0.89	2.86	3.336(4)	114.8
N(2)-H(2A)...Cl(2)	0.89	2.68	3.336(4)	114.8
N(2)-H(2A)...Cl(4)#1	0.89	2.73	3.426(5)	136.2
N(2)-H(2B)...Cl(2)#2	0.89	2.59	3.408(5)	153.8
N(2)-H(2B)...Cl(3)#3	0.89	2.83	3.383(4)	121.7
N(2)-H(2C)...Cl(3)#1	0.89	2.96	3.406(5)	112.7
N(2)-H(2C)...Cl(4)#3	0.89	2.55	3.371(4)	153.3

Symmetry transformations used to generate equivalent atoms:
#1 x+1/2, -y+1, z; #2 x-1/2, -y+1, z; #3 x, y-1, z

Table S7. Hydrogen bond lengths (Å) and angles (°) for $(4\text{-FbaH})_2CuCl_4$ at 93 K.

D-H…A	d(D-H)	d(H…A)	d(D…A)	∠(DHA)
N(1)-H(1A)...Cl(2)#3	0.91	2.54	3.315(3)	142.6
N(1)-H(1A)...Cl(2A)#3	0.91	2.57	3.293(3)	136.4
N(1)-H(1B)...Cl(2)#6	0.91	2.56	3.382(3)	150.9
N(1)-H(1B)...Cl(2A)#6	0.91	2.63	3.352(3)	137.3
N(1)-H(1B)...Cl(3)	0.91	2.67	3.397(3)	137.5
N(1)-H(1B)...Cl(3A)	0.91	2.84	3.463(9)	127.2
N(1)-H(1B)...Cl(3A)#1	0.91	2.65	3.245(8)	123.8
N(1)-H(1C)...Cl(3)#6	0.91	2.65	3.334(3)	133.2
N(1)-H(1C)...Cl(3A)#6	0.91	2.77	3.459(9)	133.2
N(1)-H(1C)...Cl(3A)#7	0.91	2.50	3.241(8)	138.3

Symmetry transformations used to generate equivalent atoms:
#1 x,-y-1/2,z; #2 x,y,z-1; #3 x+1/2,y,-z-5/2;#4 x-1/ 2,y,-z-5/2; #5 x,y,z+1; #6 x+1/2,y,-z-3/2; #7 x+1/2,-y-1/2,-z-3/2; #8 -x+1/2,-y,z+1/2

Table S8. Hydrogen bond lengths (Å) and angles (°) for $(4\text{-FbaH})_2CuCl_4$ at 173 K.

D-H…A	d(D-H)	d(H…A)	d(D…A)	∠(DHA)
N(1)-H(1A)...Cl(3)	0.91	2.55	3.314(3)	142.5
N(1)-H(1A)...Cl(3A)	0.91	2.55	3.286(3)	138.0
N(1)-H(1B)...Cl(2)	0.91	2.72	3.352(3)	127.8
N(1)-H(1B)...Cl(2A)	0.91	2.66	3.404(3)	139.8
N(1)-H(1B)...Cl(3)#4	0.91	2.57	3.383(3)	149.2
N(1)-H(1B)...Cl(3A)#4	0.91	2.63	3.342(3)	135.8
N(1)-H(1C)...Cl(1)#6	0.91	2.93	3.578(3)	129.7
N(1)-H(1C)...Cl(2)#6	0.91	2.63	3.335(3)	134.4
N(1)-H(1C)...Cl(2A)#6	0.91	2.65	3.312(3)	130.8

Symmetry transformations used to generate equivalent atoms:
#1 x, -y+1/2, z; #2 x, y, z-1; #3 x+1/2, y, -z+1/2; #4 x, y, z+1; #5 x-1/2, y, -z+1/2; #6 x-1/2, y, -z+3/2

Table S9. Hydrogen bond lengths (Å) and angles (°) for $(4\text{-FbaH})_2CuCl_4$ at 298 K.

D-H…A	d(D-H)	d(H…A)	d(D…A)	∠(DHA)
N(1)-H(1A)...Cl(2)#6	0.89	2.69	3.350(3)	132.2
N(1)-H(1A)...Cl(2A)#6	0.89	2.68	3.329(3)	131.0
N(1)-H(1B)...Cl(2)	0.89	2.83	3.379(3)	121.3
N(1)-H(1B)...Cl(2A)	0.89	2.75	3.425(3)	133.3
N(1)-H(1B)...Cl(3)#4	0.89	2.56	3.403(3)	157.2
N(1)-H(1B)...Cl(3A)#4	0.89	2.61	3.362(3)	143.2

N(1)-H(1C)...Cl(3)	0.89	2.56	3.324(3)	144.9
N(1)-H(1C)...Cl(3A)	0.89	2.58	3.291(3)	137.4

Symmetry transformations used to generate equivalent atoms:

#1 x, -y+1/2, z; #2 x, y, z-1; #3 x+1/2, y, -z+1/2; #4 x, y, z+1; #5 x-1/2, y, -z+1/2; #6 x-1/2, y, -z+3/2

Table S10. Atomic coordinates of $(2\text{-FbaH})_2CuCl_4$ at 298 K.

	x	***y***	***z***
Cu(1)	1	2	0.5000
Cl(1)	0.9869(4)	2.0767(4)	0.4290(1)
Cl(1A)	1.0400(4)	1.9874(4)	0.4293(1)
Cl(2)	0.9775(2)	1.5665(2)	0.4881(1)
Cl(2A)	0.5693(2)	1.9814(2)	0.4926(1)
F(1)	1.0824(4)	1.6443(4)	0.3395(1)
N(1)	1.4679(5)	1.4782(5)	0.4330(1)
C(1)	1.4523(5)	1.4203(5)	0.3555(1)
C(2)	1.3621(6)	1.1158(6)	0.2846(1)
C(3)	1.1999(5)	1.3072(7)	0.2946(1)
C(4)	1.2467(5)	1.4558(5)	0.3301(1)
C(5)	1.6130(5)	1.2229(6)	0.3442(1)
C(6)	1.5065(7)	1.5954(6)	0.3925(1)
C(7)	1.5675(6)	1.0708(6)	0.3091(1)

Table S11. Atomic coordinates of $(3\text{-FbaH})_2CuCl_4$ at 298 K.

	x	***y***	***z***
Cu(1)	0.6367(1)	0.7522(1)	0.4992(1)
Cl(1)	0.6273(1)	0.7169(1)	0.4292(1)
Cl(2)	0.8615(1)	0.5430(1)	0.5046(1)
Cl(3)	0.4318(1)	0.9831(1)	0.4935(1)
Cl(4)	0.6160(1)	0.7710(1)	0.5694(1)
F(1)	0.8585(5)	0.5904(5)	0.2912(1)
F(2)	0.8538(5)	-0.0875(5)	0.7085(1)
N(1)	0.6697(6)	0.2730(4)	0.4349(1)
N(2)	0.6647(6)	0.2298(5)	0.5629(2)
C(1)	0.5846(6)	0.2137(6)	0.3960(1)
C(2)	0.7016(6)	0.2377(5)	0.3594(1)
C(3)	0.7315(5)	0.4082(6)	0.3424(1)
C(4)	0.8335(7)	0.4217(9)	0.3077(1)
C(5)	0.9070(8)	0.2687(9)	0.2894(2)
C(6)	0.8779(7)	0.1033(11)	0.3065(2)

C(7)	0.7749(6)	0.0845(7)	0.3408(1)
C(8)	0.5750(6)	0.2717(7)	0.6017(1)
C(9)	0.6927(7)	0.2559(5)	0.6389(1)
C(10)	0.7248(5)	0.0872(7)	0.6563(1)
C(11)	0.8252(7)	0.0792(10)	0.6913(2)
C(12)	0.8984(9)	0.2299(10)	0.7089(2)
C(13)	0.8645(8)	0.3989(12)	0.6914(2)
C(14)	0.7624(6)	0.4110(7)	0.6563(1)

Table S12. Atomic coordinates of $(4\text{-FbaH})_2CuCl_4$ at 298 K.

	x	***y***	***z***
Cu(1)	0.8251(1)	0.2500	0.2635(1)
Cl(1)	0.8325(1)	0.3227(1)	0.2682(1)
Cl(2)	0.8131(2)	0.2500	0.6992(4)
Cl(2A)	0.8136(2)	0.2500	0.8257(4)
Cl(3)	0.6096(2)	0.2500	0.2313(4)
Cl(3A)	0.5436(2)	0.2500	0.2303(4)
F(1)	0.3634(2)	0.4875(1)	0.2428(3)
N(1)	0.5600(2)	0.3153(1)	0.7217(4)
C(1)	0.4232(3)	0.4564(1)	0.3774(5)
C(2)	0.4257(2)	0.4080(1)	0.7228(5)
C(3)	0.5416(2)	0.3927(1)	0.6434(4)
C(4)	0.5975(2)	0.4108(1)	0.4325(5)
C(5)	0.3653(3)	0.4402(1)	0.5880(6)
C(6)	0.5391(3)	0.4430(1)	0.2962(5)
C(7)	0.6045(3)	0.3576(1)	0.7907(5)

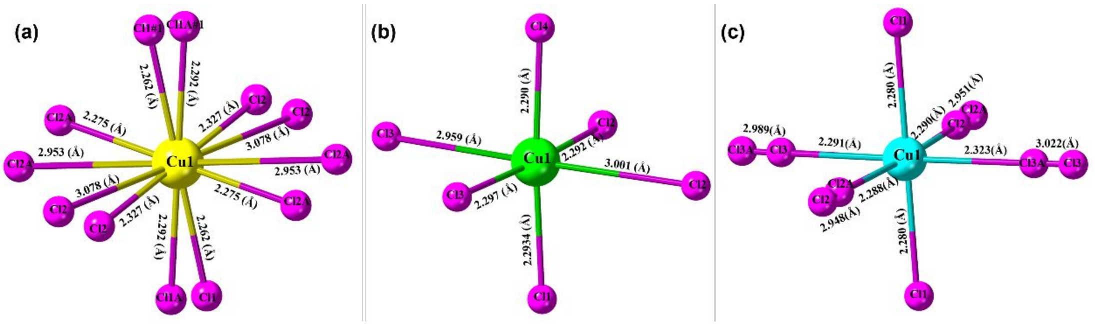

Figure S4. The nature of the distortion within the $CuCl_6$ octahedra in (a) $(2\text{-FbaH})_2CuCl_4$, (b) $(3\text{-FbaH})_2CuCl_4$, and (c) $(4\text{-FbaH})_2CuCl_4$ at 298 K.

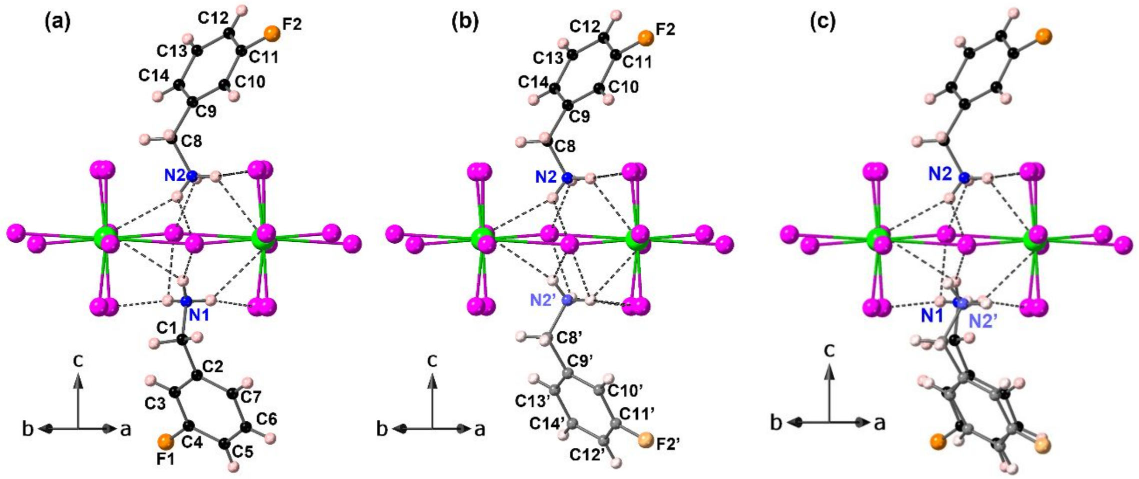

Figure S5. The symmetry breaking of $(3\text{-FbaH})_2CuCl_4$. The true structure is shown on the left in (a). The middle picture (b) shows a simulated representation of the second molecule derived by application of mirror symmetry through the Cu-Cl plane. The right-hand image (c) overlays the real structure with the structure derived in (b).

Table S13. Three-dimensional distances of the two overlapping (3-FbaH^+) molecules.

	(Å)
N1…N2’	0.317
C1…C8’	0.429
C2…C9’	0.153
C3…C13’	0.235
C4…C14’	0.287
C5…C12’	0.291
C6…C11’	0.439
C7…C10’	0.380
F1…F2’	2.347

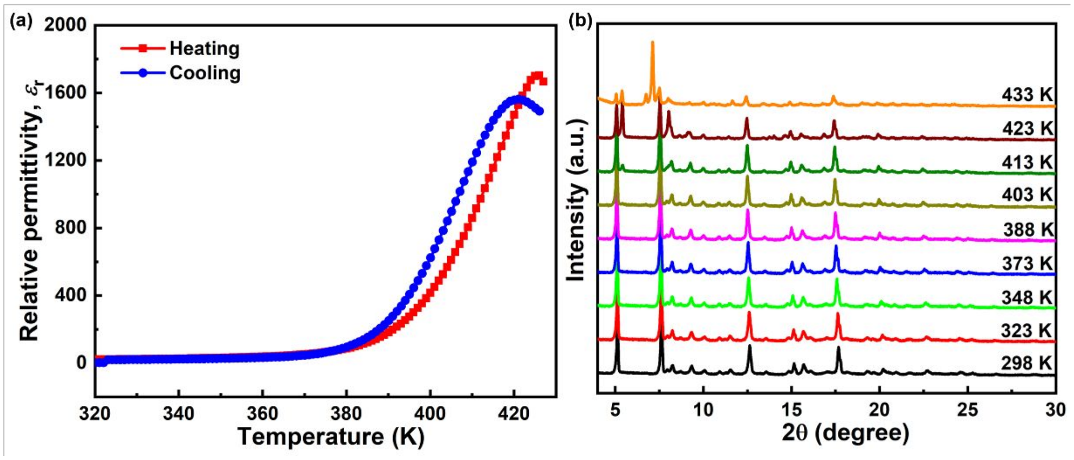

Figure S6. (a) Real part ε_r of the dielectric permittivity for $(3\text{-FbaH})_2CuCl_4$ at 100 KHz in heating and cooling runs. (b) Variable-temperature PXRD patterns from room temperature to 433 K.

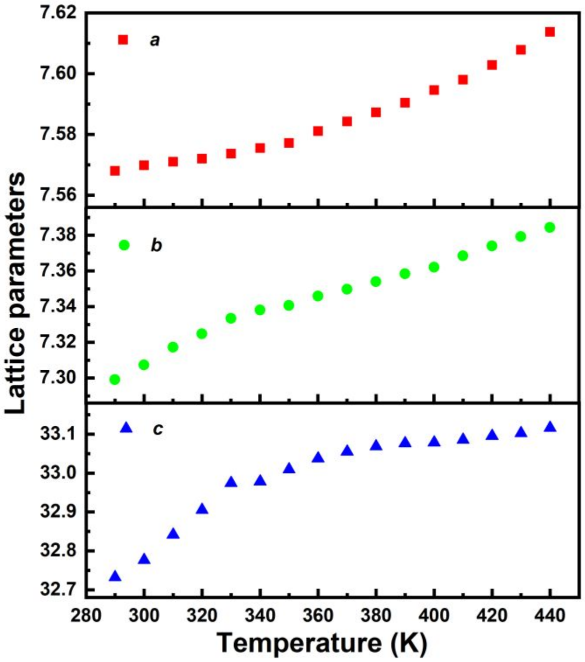

Figure S7. Thermal evolution of the lattice metrics obtained from Rietveld refinement of SXRD data for $(3\text{-FbaH})_2CuCl_4$ at the range 290 K to 440 K.

High-resolution synchrotron X-ray powder diffraction (SXRD) data for $(3\text{-FbaH})_2CuCl_4$ were collected from 290 K to 440 K at Beamline I11 Diamond Light Source, UK. Then, we performed the GSAS refinement of the SXRD data. Thermal evolutions of unit cell parameters are plotted in Figure S7. We can conclude that unit cell parameters *a*, *b*, and *c*

of $(3\text{-FbaH})_2CuCl_4$ increase upon heating, though no phase transition occurs.

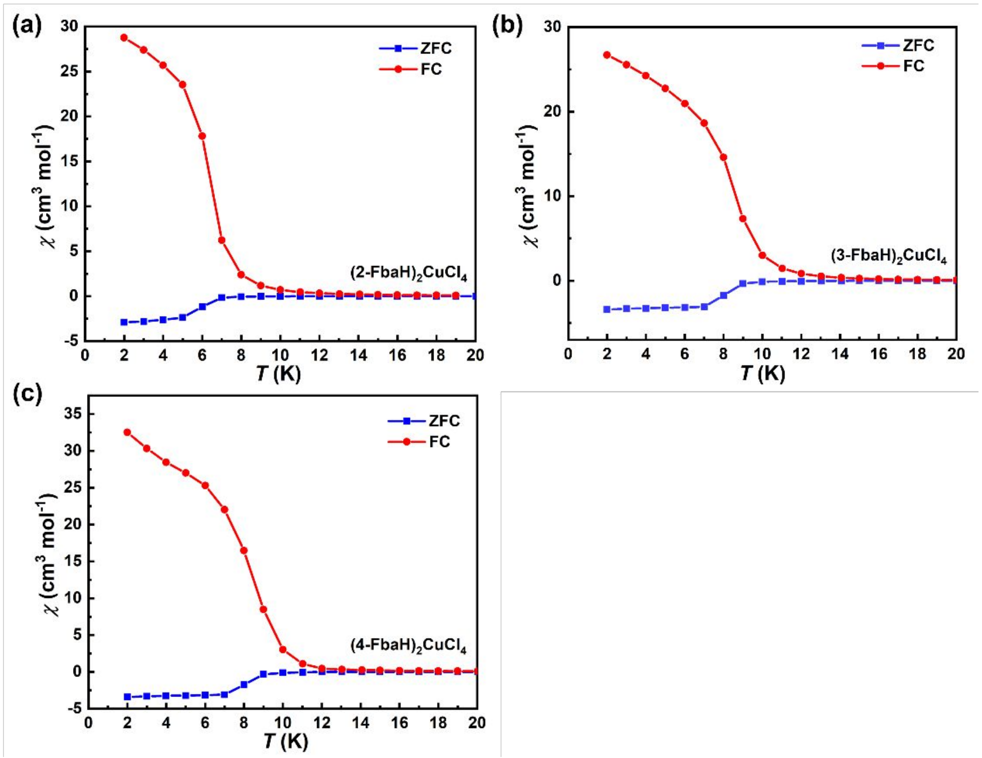

Figure S8. ZFC and FC curves at 100 Oe from 2 to 20 K for (a) $(2\text{-FbaH})_2CuCl_4$, (b) $(3\text{-FbaH})_2CuCl_4$ and (c) $(4\text{-FbaH})_2CuCl_4$.

The ZFC and FC curves measured at 100 Oe diverge at 10 K. Since for the ZFC runs, the field was simply set to zero (zero current), there remains a small amount of trapped flux inside the magnetic coils. This led to effectively a small negative applied field giving rise to a smaller spontaneous moment of opposite sign to that of the FC measurements. The data clearly show however that the magnetization is reversible at temperatures above transition temperature.

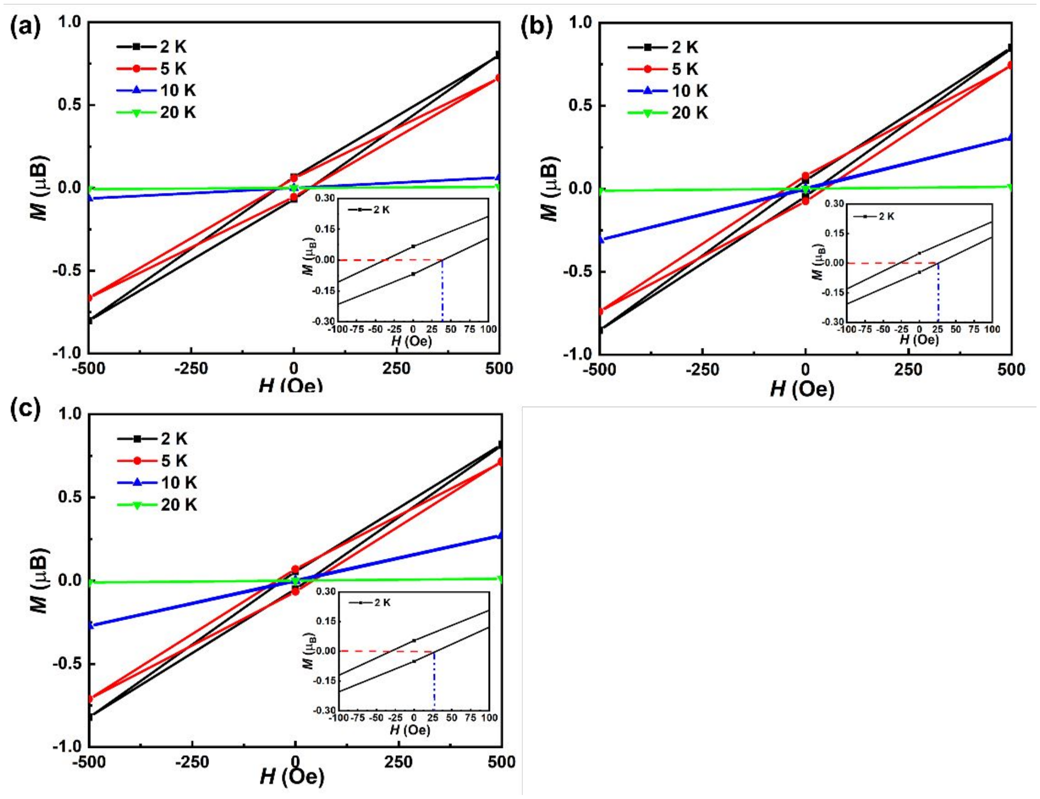

Figure S9. Magnetization (*M*) versus magnetic field (*H*) at 2, 5, 10 and 20 K between -500 to 500 Oe for (a) $(2\text{-FbaH})_2CuCl_4$, (b) $(3\text{-FbaH})_2CuCl_4$ and (c) $(4\text{-FbaH})_2CuCl_4$. Inset: the low-field region -100 to 100 Oe of the hysteresis loops at 2 K.
